# Siderophore-mediated zinc acquisition enhances enterobacterial colonization of the inflamed gut

**DOI:** 10.1038/s41467-021-27297-2

**Published:** 2021-12-01

**Authors:** Judith Behnsen, Hui Zhi, Allegra T. Aron, Vivekanandan Subramanian, William Santus, Michael H. Lee, Romana R. Gerner, Daniel Petras, Janet Z. Liu, Keith D. Green, Sarah L. Price, Jose Camacho, Hannah Hillman, Joshua Tjokrosurjo, Nicola P. Montaldo, Evelyn M. Hoover, Sean Treacy-Abarca, Benjamin A. Gilston, Eric P. Skaar, Walter J. Chazin, Sylvie Garneau-Tsodikova, Matthew B. Lawrenz, Robert D. Perry, Sean-Paul Nuccio, Pieter C. Dorrestein, Manuela Raffatellu

**Affiliations:** 1 Department of Microbiology & Molecular Genetics, University of California Irvine, Irvine, CA USA; 2 Department of Microbiology & Immunology, University of Illinois Chicago, Chicago, IL USA; 3 Division of Host-Microbe Systems & Therapeutics, Department of Pediatrics, University of California San Diego, La Jolla, CA 92093 USA; 4 Skaggs School of Pharmacy and Pharmaceutical Sciences, University of California San Diego, La Jolla, CA USA; 5 Collaborative Mass Spectrometry Innovation Center, University of California, San Diego, La Jolla, CA 92093 USA; 6 University of Kentucky PharmNMR Center, College of Pharmacy, University of Kentucky, Lexington, KY 40536-0596 USA; 7 Department of Pharmaceutical Sciences, College of Pharmacy, University of Kentucky, Lexington, KY 40536-0596 USA; 8 Department of Microbiology and Immunology, University of Louisville School of Medicine, Louisville, KY 40202 USA; 9 Department of Biochemistry and Chemistry, and Center for Structural Biology, Vanderbilt University Medical Center, Nashville, TN USA; 10 Department of Pathology, Microbiology, and Immunology, Vanderbilt University Medical Center, Nashville, TN USA; 11 Center for Predictive Medicine for Biodefense and Emerging Infectious Diseases, Department of Microbiology and Immunology, University of Louisville School of Medicine, Louisville, KY 40202 USA; 12 Department of Microbiology and Immunology, University of Kentucky, Lexington, KY 40536 USA; 13 Center for Microbiome Innovation, University of California San Diego, La Jolla, CA 92093 USA; 14 Chiba University-UC San Diego Center for Mucosal Immunology, Allergy, and Vaccines (CU-UCSD cMAV), La Jolla, CA 92093 USA

**Keywords:** Bacterial pathogenesis, Small molecules, Bacterial physiology, Metals

## Abstract

Zinc is an essential cofactor for bacterial metabolism, and many *Enterobacteriaceae* express the zinc transporters ZnuABC and ZupT to acquire this metal in the host. However, the probiotic bacterium *Escherichia coli* Nissle 1917 (or “Nissle”) exhibits appreciable growth in zinc-limited media even when these transporters are deleted. Here, we show that Nissle utilizes the siderophore yersiniabactin as a zincophore, enabling Nissle to grow in zinc-limited media, to tolerate calprotectin-mediated zinc sequestration, and to thrive in the inflamed gut. We also show that yersiniabactin’s affinity for iron or zinc changes in a pH-dependent manner, with increased relative zinc binding as the pH increases. Thus, our results indicate that siderophore metal affinity can be influenced by the local environment and reveal a mechanism of zinc acquisition available to commensal and pathogenic *Enterobacteriaceae*.

## Abstract (web summary)

Zinc is an essential cofactor for bacterial metabolism. Here, the authors show that the probiotic bacterium *Escherichia coli* Nissle 1917 utilizes the siderophore yersiniabactin as a zincophore, allowing the microbe to grow in zinc-limited media and to thrive in the inflamed gut.

## Introduction

The *Enterobacteriaceae* are a diverse family of bacteria that inhabit the gastrointestinal tract. Members of this group include the enteric pathogen *Salmonella enterica* serovar Typhimurium (*S*. Typhimurium, or STm), as well as *Escherichia coli*, a species that comprises myriad commensals, pathobionts, and pathogens. Both STm and *E. coli* can colonize the intestine of mammals and thrive in inflammatory conditions^1–5^. During homeostasis, the gut microbiota is primarily composed of obligate anaerobes belonging to the phyla Bacteroidetes and Firmicutes^6^. In the inflamed gut, however, the oxidative environment suppresses obligate anaerobes and favors the growth of facultative anaerobes, which include pathogenic and commensal *Enterobacteriaceae*^1,2,4,5,7,8^.

One mechanism that enables enterobacterial growth in the inflamed gut is the ability to scavenge metal nutrients. Many biological processes including DNA replication, transcription, respiration, and oxidative stress responses require iron, manganese, cobalt, nickel, copper, and/or zinc^9^. Iron is one of the most abundant transition metal ions in living organisms, and serves as an essential cofactor in central metabolism and respiration^10,11^. The other most abundant metal ion is zinc, which is a cofactor for an estimated 5–6% of all proteins^12^, and whose functions include acting as the catalytic center in enzymes such as metalloproteases, superoxide dismutases, and metallo-β-lactamases. Thus, bacteria must be able to acquire sufficient amounts of both iron and zinc in order to survive and replicate in a given environment.

Bacteria living inside the human host face particular difficulties in obtaining these metal nutrients. During homeostasis, the availability of such metal ions is actively limited by the host and by the resident microbiota. Moreover, nutrient metal availability is further restricted during inflammation in a process termed “nutritional immunity”^13^, wherein the host secretes antimicrobial proteins that sequester iron, zinc, and manganese from microbes to limit their growth. We have previously shown that the pathogen STm overcomes host nutritional immunity by obtaining iron, zinc and manganese in the inflamed gut^1,14–16^. In response to iron limitation, STm secretes enterobactin and salmochelin, which are small iron-scavenging molecules called siderophores^17,18^. In response to zinc limitation, STm expresses the high-affinity zinc transporter ZnuABC^15,19,20^. STm also expresses the ZupT permease, which transports zinc and other divalent metal ions^21,22^. Independently, each of these transporters has been shown to contribute to STm virulence in mouse models of infection^15,19,20,23,24^.

High-affinity zinc acquisition systems enable microbes to overcome zinc sequestration by the host protein calprotectin (CP), a heterodimer of the S100A8 and S100A9 proteins^25^. CP constitutes up to 40% of neutrophil cytosolic content^26^, and the expression of its two subunits can be induced in epithelial cells following stimulation with IL-17 and IL-22^1,27^. In the inflamed gut, expression of ZnuABC enables STm to overcome CP-mediated zinc sequestration, outcompete the microbiota, and colonize to high levels^1,15^.

In addition to STm, other *Enterobacteriaceae* can thrive in the inflamed intestine. One such example is the probiotic bacterium *Escherichia coli* Nissle 1917 (*E. coli* Nissle, or EcN), a strain that was first isolated in WWI from the stool of a soldier who did not develop gastroenteritis during a *Shigella* outbreak^28^. Since then, EcN has proven to be effective in the treatment and prevention of intestinal disorders including chronic constipation, ulcerative colitis, and infantile diarrhea^29–32^, albeit its mechanisms of action are not well understood. Our previous work has demonstrated that EcN reduces STm colonization in mouse models of gastroenteritis by utilizing multiple iron uptake systems and by secreting antimicrobial proteins known as microcins to outcompete the pathogen^3,33^.

In this work, we show that an EcN strain lacking ZnuABC and ZupT is still able to grow appreciably in zinc-limited media, leading us to discover that EcN expresses an additional means of acquiring zinc. Using our recently developed native spray metabolomics approach^34^, we find that the siderophore yersiniabactin (Ybt) produced by EcN is capable of binding zinc, and that EcN utilizes Ybt as a zincophore. Moreover, we demonstrate that EcN utilizes Ybt, in addition to the zinc transporters ZnuABC and ZupT, to effectively acquire zinc in vitro, to tolerate the antimicrobial activity of CP, and to colonize the inflamed gut.

## Results

### *E. coli* Nissle is more resistant to calprotectin-mediated zinc sequestration than *S*. Typhimurium

**Fig. 1:**
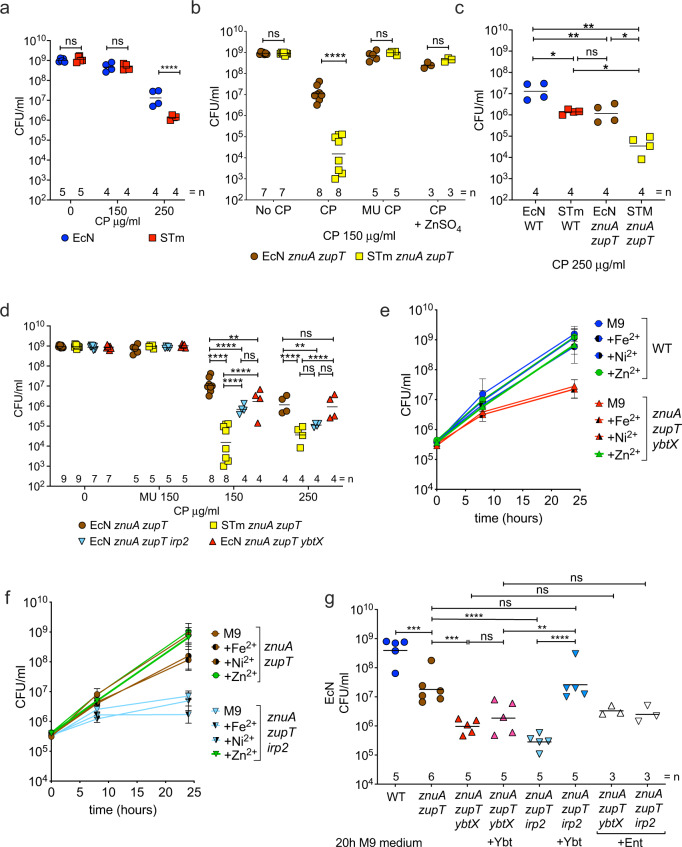
*E. coli* Nissle resistance to calprotectin-mediated zinc limitation in vitro is dependent on ZnuABC, ZupT, and yersiniabactin. **a** *E. coli* Nissle (EcN) and *S*. Typhimurium (STm) wild-type were grown in modified LB medium without calprotectin (CP), or supplemented with 150 µg/ml or 250 µg/ml CP. Statistics: two-way ANOVA with Šidák’s multiple comparisons test. **b** EcN and STm *znuA zupT* mutants were grown in modified LB medium without CP (No CP), or supplemented with 150 µg/ml CP, 150 µg/ml Site I/II knockout mutant CP (MU CP), or 150 µg/ml CP plus 5 µM ZnSO_4_ (CP + ZnSO_4_). Statistics: two-way ANOVA with Šidák’s multiple comparison test. **c** EcN and STm wild-type and *znuA zupT* mutants were grown in modified LB medium supplemented with 250 µg/ml CP. Statistics: one-way ANOVA with Tukey’s multiple comparisons test. **d** EcN and STm *znuA zupT* mutants, as well as EcN triple mutants (*znuA zupT irp2*; *znuA zupT ybtX*), were grown in modified LB without CP, or supplemented with either 150 µg/ml mutant CP (MU 150), or with 150 µg/ml (150) or 250 µg/ml CP (250). Statistics: two-way ANOVA with Tukey’s multiple comparisons test. **e**, **f** EcN wild-type, double (*znuA zupT*) and triple (*znuA zupT irp2*; *znuA zupT ybtX*) mutants were grown in M9 medium (*n* = 3 biologically independent replicates) or in M9 supplemented with either 5 µM ZnSO_4_ (*n* = 3 biologically independent replicates), 5 µM FeCl_2_ (*n* = 5 biologically independent replicates), or 5 µM NiCl_2_ (*n* = 5 biologically independent replicates). **g** EcN wild-type and indicated double and triple mutants were grown in M9 medium or in M9 supplemented with either 1 µM yersiniabactin (Ybt) or enterobactin (Ent). Statistics: one-way ANOVA with Tukey’s multiple comparisons test. **a**–**g** Growth was quantified by enumeration of bacterial CFU on selective media after **a**–**d** 16 h static, **e**, **f** 8 h and 24 h shaking, or **g** 20 h shaking incubation. Data are representative of three independent experiments. **a**–**d**, **g** Bars represent the geometric mean. The number (=n) of biologically independent replicates for each group is indicated in each figure panel. **e**, **f** Data are presented as geometric mean values ± geometric SD. *P value ≤ 0.05; **P value ≤ 0.01; ***P value ≤ 0.001, ****P value ≤ 0.0001; ns not significant. Exact *P* values are reported in Supplementary Data 2. Source data are provided as a Source Data file.

We have previously shown that multiple iron uptake systems enable EcN to colonize the inflamed gut and to compete with STm^33^. As zinc is also limited in the inflamed gut, we hypothesized that EcN must also have robust mechanisms for acquiring this metal. We thus compared the growth of EcN to the growth of STm in a rich medium supplemented with CP, a host antimicrobial protein that sequesters zinc and limits its availability to microbes^15,25^. To this end, we employed CP concentrations (125–250 μg/ml) comparable to those found in the inflamed gut^15^. EcN and STm showed similar growth in rich media without the addition of CP, but EcN grew significantly better than STm in media supplemented with CP (Fig. 1a and Supplementary Fig. 1a–c). Thus, EcN is more resistant than STm to the antimicrobial activity of CP in vitro and prompted us to investigate the underlying mechanism.

Both EcN and STm encode two known zinc transport systems: the high-affinity zinc transporter ZnuABC and the permease ZupT^15,19,20,23,24^. Although the function of these two transporters in EcN has not been directly investigated, their disruption significantly diminishes the capacity of the closely-related uropathogenic *E. coli* strain CFT073 to grow in zinc-depleted culture media and to cause urinary tract infection^35^. To determine whether the difference in CP-resistance between EcN and STm is the result of variations related to ZnuABC and ZupT, we disrupted these transporters in both EcN and STm by deleting the genes *znuA* and *zupT*. As expected, both mutant strains (EcN *znuA zupT* and STm *znuA zupT*) grew slower than their respective parental strains in the presence of CP, but not in the presence of a Site I/II knockout mutant CP (MU CP; lacks the ability to bind zinc)^36,37^, or when ZnSO_4_ was added to the media (Fig. 1b and Supplementary Fig. 1d, e). These results indicated that ZnuABC and ZupT have similar functions in both EcN and STm, and mediate evasion of CP-dependent antimicrobial activity.

Puzzlingly, we observed that the EcN *znuA zupT* mutant grew up to 1000-fold better than the STm *znuA zupT* mutant in the presence of 125–150 μg/ml CP (Fig. 1b and Supplementary Fig. 1e). Although higher concentrations of CP (250 μg/ml) reduced the growth of the EcN *znuA zupT* mutant, it was still 100-fold higher than the STm *znuA zupT* mutant (Fig. 1c). As the addition of ZnSO_4_ rescued the growth of both the EcN and STm *znuA zupT* mutants (Supplementary Fig. 1f), we posited that EcN acquires zinc via an additional mechanism absent in STm.

### A product of the yersiniabactin operon promotes zinc acquisition by *E. coli* Nissle in zinc-limited media

In iron-limiting conditions, EcN acquires iron by producing the siderophores enterobactin, salmochelin, aerobactin, and yersiniabactin (Ybt)^33^. Although the importance of siderophores in scavenging iron has been well-demonstrated in biological systems, chemists have known for decades that some siderophores can bind other metals besides iron (reviewed in Johnstone and Nolan^38^). Among the siderophores produced by EcN, Ybt has been shown to also bind copper, gallium, nickel, cobalt, and chromium^39^. Intriguingly, a product of the Ybt gene cluster has been proposed to contribute to zinc acquisition by the pathogen *Yersinia pestis*^40,41^; however, its identity and mechanism are unknown, as two prior studies did not provide evidence of direct zinc binding by Ybt^39,41^. We thus sought to determine whether, in addition to ZnuABC and ZupT, EcN uses a product of the Ybt operon to acquire zinc under zinc-limiting conditions.

To this end, we deleted the *ybt* cluster’s *irp2* gene that encodes the synthetase HMWP2, thus rendering EcN unable to synthesize Ybt^42–44^. We also deleted the *ybtX* gene, which encodes for an inner membrane permease that, in *Y. pestis*, was found to be dispensable for iron uptake, but required for zinc uptake, as a *znu ybtX* mutant is unable to grow in zinc-limited medium^40,41^. Of note, the first published genome sequence of EcN wild-type (GenBank CP007799.1, Reister et al.^45^) indicated that *irp1* and *irp2* were disrupted (frameshifted and insertion sequence, respectively), although a recent sequencing effort utilizing our lab’s EcN wild-type strain revealed these genes to be intact (GenBank “CP022686.1”), which is consistent with a prior study showing that EcN produces Ybt^46^. Next, we tested the growth of EcN strains lacking these genes, in addition to the *znuA zupT* genes, in metal-limiting conditions (M9 minimal medium). Strains lacking *znuA zupT* and either *irp2* or *ybtX* displayed a severe growth defect in M9 minimal medium, where the strains grew 1000-fold less than EcN wild-type and more than 10-fold less than EcN *znuA zupT* (Fig. 1d). Furthermore, growth of all mutants was restored in ZnSO_4_-supplemented M9 minimal medium (Supplementary Fig. 1g) and in LB broth without metal limitation (Supplementary Fig. 1h). Moreover, only zinc supplementation, but not iron or nickel supplementation, restored growth of the mutants to wild-type levels (Fig. 1e, f), confirming that the observed growth defects of the mutants were indeed due to zinc deficiency. Taken together, these results suggested that a product of the *ybt* gene cluster contributes to zinc acquisition by the probiotic EcN in zinc-limited media. We therefore hypothesized that the Ybt locus may encode for the production of a zincophore.

### Yersiniabactin is a zincophore

To identify whether the *ybt* gene cluster produces a zincophore, we cultured EcN wild-type and the *irp2* mutant in M9 minimal media and collected culture pellets and supernatants to then run ultra-high performance liquid chromatography tandem mass spectrometry (UHPLC-MS/MS). In addition to running UHPLC-MS/MS metabolomics on these samples, we performed experiments using post-liquid chromatography (LC) pH adjustment to 6.8 and infusion of a zinc acetate solution, followed by mass spectrometry – a workflow that we term native electrospray metabolomics – in order to assess whether any of the metabolites produced were capable of binding zinc^34^. This native metabolomics strategy is then combined with ion identity-based molecular networking, a new computational and data visualization strategy that allows for the discovery of mass spectrometry features with the same retention time and specified mass offsets^34^; mass spectrometry features with the same retention time and a mass difference resulting from zinc binding can be discovered directly from complex metabolomics samples.

**Fig. 2:**
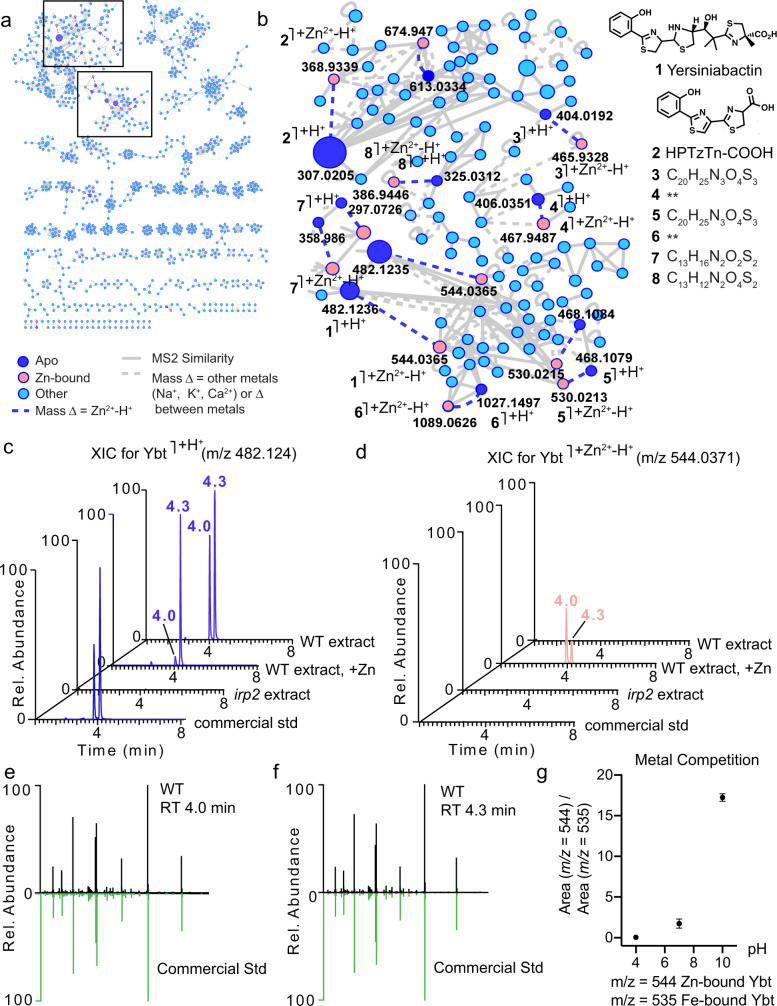
Yersiniabactin is produced by *E. coli* Nissle and directly binds zinc in a pH-dependent manner. **a**, **b** Native spray metal metabolomics was used to identify zinc-binding small molecules present in isolated EcN supernatant extracts. Zinc-binding molecules, including Ybt and other truncations, are concentrated in the boxed molecular families enlarged in panel **b**. Zinc-binding small molecules are observed when post-LC infusion of Zn^2+^ and post-pH adjustment are performed. Zinc-bound molecules are shown in salmon, while the corresponding protonated (Apo) form of these molecules is shown in dark blue. Structures and molecular formulas (generated using SIRIUS 4.0^78^) are provided. **c** Extracted ion chromatogram (XIC) for apo-Ybt ([M + H^+^]^+^ = 482.1236) is observed as two peaks (present at 4.0 and 4.3 m) in wild-type EcN supernatant (WT extract); tautomerization occurring at C10 results in the racemic mixture (Fig. 3). When post-LC pH adjustment and Zn^2+^-infusion are performed, the majority of the peak at 4.3 remains apo-Ybt (WT extract, +Zn). Neither apo-Ybt peak is present in the EcN *irp2* knockout supernatant (*irp2* extract). Commercial Ybt (*commercial std*) also elutes with a minor peak at 4.0 and a major peak at 4.3 m. **d** Zn^2+^-bound Ybt ([M + Zn^2+^-H^+^]^+^ = 544.0371) is not observed in the XIC of wild-type samples (WT extract), in *irp2* knockout samples (*irp2* extract), or in the commercial standard (*commercial std*) when the standard LC-MS/MS method is applied; however, when native spray metal metabolomics is applied (WT extract, +Zn; post-LC infusion of Zn^2+^ in conjunction with pH neutralization), Zn^2+^-bound Ybt is observed ([M + Zn^2+^-H^+^]^+^ = 544.0371) as the major species in the first peak (at retention time = 4.01 m). **e**, **f** Mirror plots show that peaks present at **e** 4.0 min and **f** 4.3 min (black; MS/MS of [(M + 2H^+^)/2 = 241.5654) both match the MS/MS from commercial Ybt standard (green). **g** Metal competition data for direct injection experiments run at pH 4, 7, and 10, in which Ybt was added to buffer in the presence of both iron and zinc; *n* = 3 biologically independent replicates). The ratio of extracted peak area of Zn^2+^-bound Ybt ([M + Zn^2+^-H^+^]^+^ = 544.0371) to extracted peak area of Fe^3+^-bound Ybt ([M + Fe^3+^-H^+^]^+^ = 535.0351) is shown at each of the three tested pH values.

**Fig. 3:**
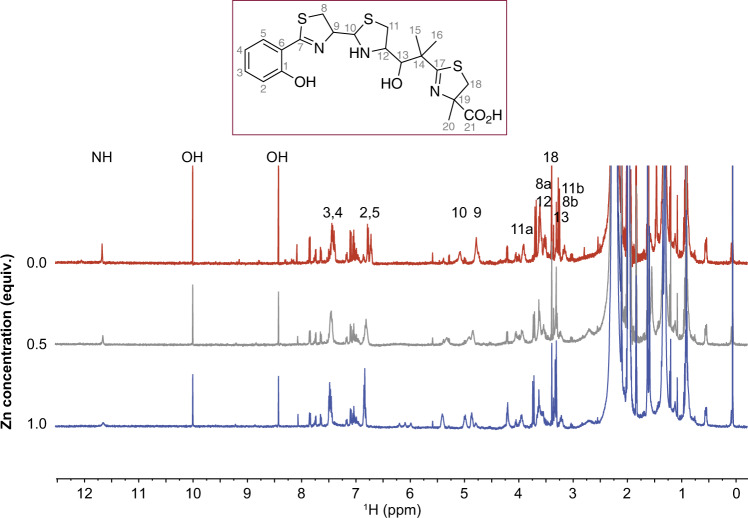
1D 1H NMR confirms direct zinc binding to yersiniabactin. 1D ^1^H NMR spectra of Ybt dissolved in CD_3_CN (top trace, 0 equivalents of Zn, red trace) as increasing zinc is titrated into the solution (0.5 equiv., gray trace), (1.0 equiv., blue trace). The loss of intensity of the NH and OH signals arises from the coordination of zinc by the corresponding N and O atoms; the signals that shift correspond to protons whose electronic environment changes due to binding of the Zn^2+^ ion. Only partial binding is observed because Ybt is in rapid equilibrium between two tautomers at C10 in addition to hydrolysis that occurs at C10 ^49^.

Using this native electrospray metabolomics workflow, a number of zinc-binding small molecules were observed in the wild-type supernatant samples (Fig. 2a, b); zinc-bound nodes (each node represents an MS1 feature and its clustered MS/MS spectra) are shown in salmon and are connected to protonated nodes (dark blue) with a blue dashed line (indicating a *m/z* delta = [Zn^2+^-H^+^]^+^) (Fig. 2a, b). Furthermore, two peaks were observed in culture supernatant (Fig. 2c) from EcN wild-type that were absent in the *irp2* mutant cultured in M9 minimal media (Fig. 2c**)**. Feature-based molecular networking using MZmine in conjunction with Global Natural Products Social (GNPS) Molecular Networking^47,48^ allowed us to putatively identify these two peaks as Ybt. Ybt is known to tautomerize at C10 (Fig. 3), so exists as two diastereomers^49^. We confirmed that these peaks were two diastereomers of Ybt by matching the retention time, exact mass, and MS/MS spectra acquired from culture extracts to an authentic Ybt standard (Fig. 2e, f). Post-LC pH neutralization and zinc-infusion revealed the zinc-bound Ybt species, indicating that Ybt is indeed capable of binding zinc (Fig. 2d). To our surprise, we also found that one of the diastereomers (at retention time = 4.0 min) seems to bind zinc with higher preference than the other (at retention time = 4.3 min) (Fig. 2d). Since Ybt was initially discovered as an iron-binding molecule, and thus termed a siderophore, we next sought to determine the preferential conditions for binding iron versus zinc. To assess the competition between iron and zinc binding, we performed direct infusion mass spectrometry competition experiments at multiple pH values. In these experiments, we added equimolar amounts of zinc and iron to Ybt in ammonium acetate buffer adjusted to pH 4, 7, and 10. While Ybt preferentially binds iron at low pH (pH 4), it exhibits a higher preference for zinc at high pH (pH 10) (Fig. 2g). These experiments suggest that Ybt-Zn^2+^ binding is most competitive to ferric iron binding at basic pH; however, another possible explanation is that less ferric iron than Zn^2+^ is available to bind at basic pH due to solubility differences between the two metals. Nevertheless, at neutral pH (pH 7), Ybt was observed bound to iron or zinc at roughly equal proportion (Fig. 2g).

To confirm the zinc-binding observed by native electrospray metabolomics, we monitored a zinc-titration into Ybt by 1D ^1^H NMR (Fig. 3). Although Ybt is in equilibrium between two tautomers at C10 that seem to have different relative affinities for zinc, only one set of signals is observed in the spectra. This same observation was reported in earlier studies of gallium binding to Ybt^49^. The addition of zinc modulates a number of signals, including those of the NH proton and the two hydroxyl proton peaks at ~11.6, 10.0, and 8.4 ppm. The intensity of these well-resolved peaks decreases progressively upon addition of 0.5 and 1.0 equivalents of zinc, which is consistent with the nitrogen (N10-12) and oxygen (O1 and O13) heteroatoms chelating the zinc atom (Fig. 3), in a manner similar to Ybt binding of iron^50^ and copper^51^, and zinc binding by the *Pseudomonas* sp.-derived compound micacocidin A^52^. Finally, we found that increasing the pH of the solution via addition of 0–5 molar equivalents of NaOD had little effect on the NMR spectrum of zinc-bound Ybt complex, aside from the exchange of labile hydrogens with deuterium (Supplementary Fig. 2), showing that zinc is bound in the same manner across a broad range of pH. Given that zinc-Ybt complexes were observed using native electrospray metabolomics, and this was confirmed by NMR, we next assessed Ybt-zinc binding in the presence of CP. In competition experiments, we observed that CP exchanges zinc with Ybt even when Ybt-zinc was pre-formed, while the Site I/II knockout mutant CP (MU CP) does not participate in this exchange (Supplementary Fig. 3). Thus, CP can outcompete Ybt for zinc, consistent with the results from growth assays with WT and MU CP (Fig. 1). However, this does not rule out the possibility that Ybt may be able to scavenge the remaining zinc that is not bound by CP, akin to other zinc acquisition systems in bacteria.^53^

Having now discovered that Ybt can bind zinc in a physiologically relevant pH range, even in the presence of iron, we next tested whether the addition of exogenous Ybt could rescue the growth of an EcN strain that is highly susceptible to zinc limitation due to mutations in ZnuABC, ZupT, and Ybt synthesis (*znuA zupT irp2* mutant). Consistent with our hypothesis, supplementation of M9 minimal media with 1 μM purified apo-Ybt (Ybt not bound to iron) rescued the growth of the EcN *znuA zupT irp2* mutant to similar levels as the *znuA zupT* mutant (Fig. 1g). Furthermore, the growth of a strain deficient in the putative zinc-transporting inner membrane protein YbtX (*znuA zupT ybtX*) was not significantly rescued by exogenous apo-Ybt (Fig. 1g). Addition of the siderophore apo-enterobactin, which is not expected to bind to zinc, did not significantly rescue the growth of either the *znuA zupT irp2* mutant or the *znuA zupT ybtX* mutant (Fig. 1g). Thus, our results demonstrate that Ybt binds to both iron and zinc, that metal binding can be influenced by pH, and that Ybt can scavenge zinc for EcN in zinc-limited media. Next, we assessed whether Ybt enables EcN to evade the host response.

### *E. coli* Nissle’s higher resistance to calprotectin is due to yersiniabactin-mediated zinc acquisition

In the host, zinc limitation is largely dependent on the antimicrobial protein CP^54^. We thus tested whether Ybt-mediated zinc acquisition enhances EcN’s growth in CP-supplemented rich media. Above, we demonstrated that when the ZnuABC and ZupT transporters were deleted (*znuA zupT* mutants), EcN grew better than STm (Fig. 1b–d). When either *irp2* or *ybtX* were additionally deleted in EcN, growth of the *znuA zupT irp2* and the *znuA zupT ybtX* mutants were ~8-fold lower than the parental EcN *znuA zupT* strain in the presence of 150 µg/ml CP (Fig. 1d). Although the growth of EcN *znuA zupT* was further diminished in the presence of 250 µg/ml CP, the growth of the EcN *znuA zupT irp2* mutant was again ~10-fold lower, and now comparable to that of the STm *znuA zupT* mutant (Fig. 1d). These results are consistent with Ybt scavenging zinc for EcN when the metal is limited by CP. Because growth of the EcN *znuA zupT ybtX* mutant was similar to the *znuA zupT* mutant in media supplemented with 250 µg/ml CP, it is possible that zinc-bound Ybt can also be internalized via a YbtX-independent mechanism. To confirm that the growth defect of the EcN *znuA zupT irp2* mutant is due to zinc chelation by CP, we supplemented the medium with 150 µg/ml of CP Site I/II knockout mutant (Fig. 1d), or with 150 µg/ml CP and 5 µM ZnSO_4_ (Supplementary Fig. 1f). In both experiments, all strains grew to the same level. Taken together, these results indicate that Ybt-mediated zinc acquisition enhances EcN resistance to zinc limitation induced by CP and provide a mechanistic explanation for EcN’s heightened resistance to zinc limitation relative to STm.

### Yersiniabactin enhances *E. coli* Nissle colonization of the inflamed gut

**Fig. 4:**
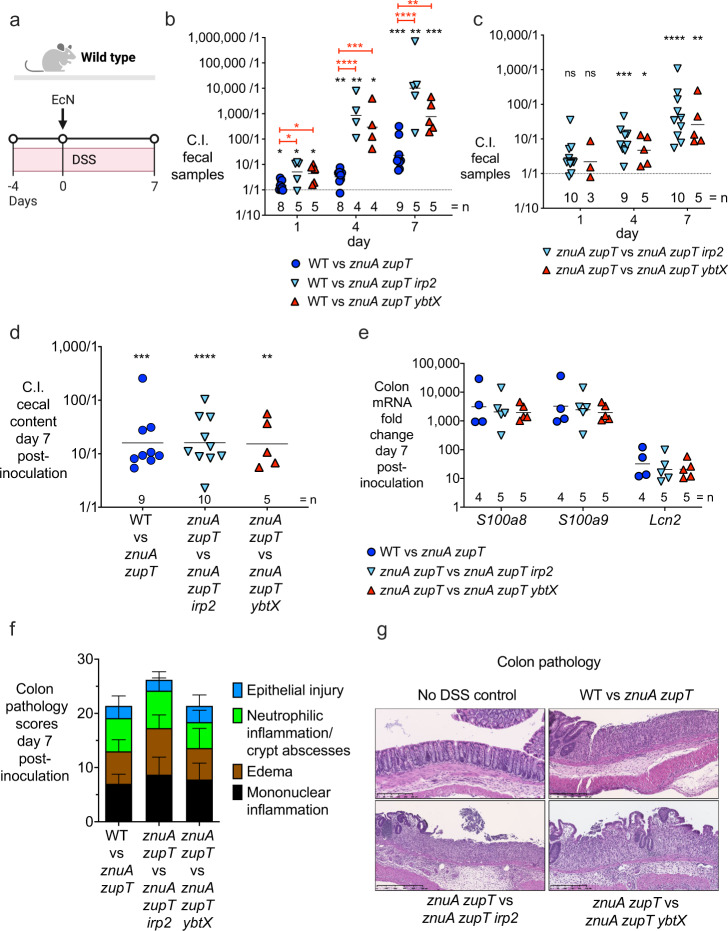
The ability to acquire zinc via yersiniabactin enhances *E. coli* Nissle colonization of the inflamed gut. **a** Experiment timeline for the DSS-induced colitis model and the administration of EcN strains. 8–10-week-old C57BL/6 female mice were given 4% (w/v) DSS in the drinking water for 4 days (day −4 to 0). On day 0, mice were orally gavaged with 1 × 10^9^ CFU of a 1:1 mixture of EcN strains. **b**, **c** Fecal samples were collected on day 1, 4, and 7, and the competitive index (C.I.) was calculated by dividing the output ratio. **b** CFU of wild type or **c** of the *znuA zupT* strain / CFU of the competing double or triple mutant strain in each group) by the CFU-enumerated input ratio of the strains. Two-sided one-sample *t* test was used on log-transformed data to accept or reject null hypothesis (theoretical mean = 0) (black stars). Statistical significance between groups was determined by one-way ANOVA and Dunnett’s multiple comparisons test of log-transformed data (red stars). **d** Cecal content was collected on day 7 and the C.I. of strains in the indicated groups was calculated as described in **b**. Two-sided one-sample *t* test was used on log-transformed data to accept or reject null hypothesis (theoretical mean = 0). **e** mRNA expression of *S100a8*, *S100a9*, and *Lcn2* was measured in the colon of mice in panel **c**. **f** Colon pathology score of mice in panel **d**, with sub-scores of each criterion (WT vs *znuA zupT* *n* = 8 samples; WT vs *znuA zupT irp2* *n* = 10 samples; WT vs *znuA zupT ybtX* *n* = 5 samples; all colon samples are biologically independent). **g** Representative stained sections (H&E, original magnification ×10, scale bars represent 250 μm) of distal colon from healthy or DSS-treated mice administered with different groups of EcN; sample size as in **f**. **b**–**e** Each data point represents a single mouse (biologically independent samples), and bars represent the geometric mean. The number (=n) of biologically independent replicates for each group is indicated in each figure panel. **f** Data are presented as mean values  ±SD. *P value ≤ 0.05; **P value ≤ 0.01; ***P value ≤ 0.001; ****P value ≤ 0.0001; ns not significant. Exact *P* values are reported in Supplementary Data 2. Source data are provided as a Source Data file.

After demonstrating that Ybt promotes EcN resistance to CP in vitro, we next sought to investigate whether Ybt confers a growth advantage to EcN during inflammatory conditions in vivo, where CP is highly expressed^1,15^ and zinc is limited^15^. To induce intestinal inflammation, we employed the dextran sodium sulfate (DSS) mouse colitis model (Fig. 4a). After 4 days of DSS administration, we orally inoculated the mice with a 1:1 mixture of EcN wild-type and *znuA zupT*, or of EcN wild-type and one of the EcN triple mutants (*znuA zupT irp2* or *znuA zupT ybtX*). EcN wild-type exhibited a significant competitive advantage over all of the mutants beginning at day 1 post-inoculation, which substantially increased by day 7, particularly for the triple mutants (Fig. 4b, d and Supplementary Fig. 4a–c). Specifically, at day 7 EcN wild-type outcompeted the *znuA zupT* mutant an average of ~23-fold, the *znuA zupT irp2* mutant an average of ~13,684-fold, and the *znuA zupT ybtX* mutant an average of ~767-fold. These results indicated that ZnuABC and ZupT are needed for optimal colonization of the inflamed gut, and that deletion of Ybt genes exacerbates the growth defect of the *znuA zupT* mutant in vivo. To further probe the specific role of Ybt, we performed a second set of competitive experiments, in which we orally inoculated DSS-treated mice with a 1:1 mixture of EcN *znuA zupT* and one of the EcN triple mutants (*znuA zupT irp2* or *znuA zupT ybtX*). Here, EcN *znuA zupT* showed a significant competitive advantage over both triple mutants, which increased over time up to ~26-fold (*znuA zupT ybtX* mutant) and ~45-fold (*znuA zupT irp2* mutant) (Fig. 4c, d). In both cases, the increased competitive advantage was due to the decreased colonization level of the triple mutants, as the *znuA zupT* mutant colonized at similar levels (Supplementary Fig. 4d, f). Of note, host antimicrobial gene expression levels (*Lcn2*, *S100a8, S100a9*) were similarly upregulated in all DSS-treated mice (Fig. 4e), and all DSS-treated mice developed similar levels of colitis, as shown by histopathology evaluation of the distal colon (Fig. 4f, g). Collectively, these results indicate that both Ybt production (via Irp2) and Ybt transport (via YbtX) enhance EcN colonization of the inflamed gut. Because Ybt production and acquisition conferred a colonization advantage to the *znuA zupT* mutant, these data support the idea that Ybt can scavenge zinc in vivo, in zinc-limited conditions such as those found in the inflamed gut.

### Inflammation and calprotectin are necessary for yersiniabactin to enhance gut colonization by *E. coli* Nissle

**Fig. 5:**
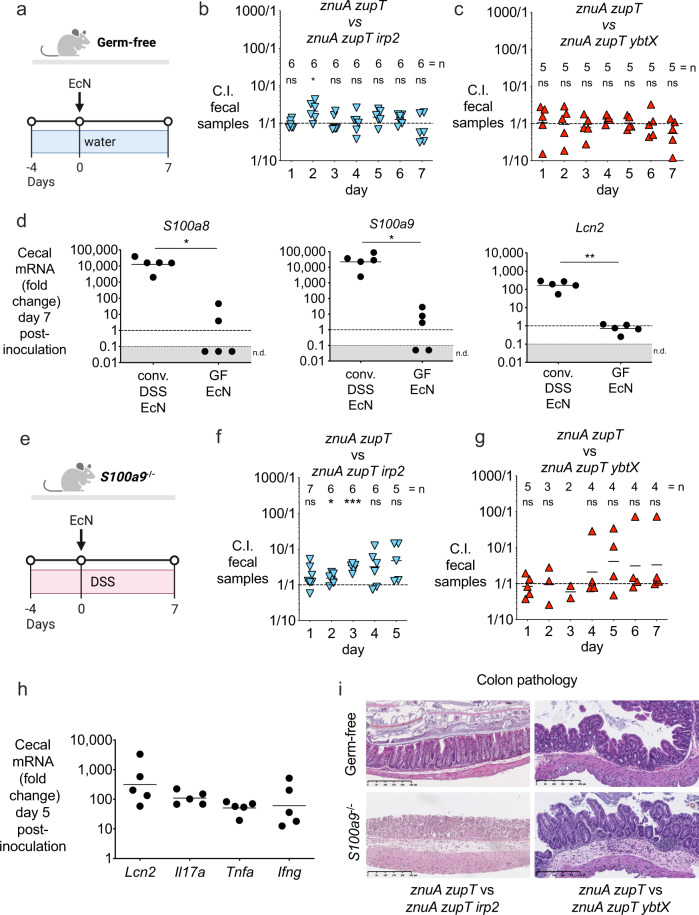
Yersiniabactin-mediated zinc acquisition provides a competitive advantage for *E. coli* Nissle in the presence of inflammation and calprotectin. **a** Experiment timeline for panels **b** and **c**. **b**, **c** Female Germ-free Swiss Webster mice were colonized with 1×10^9^ CFU of a 1:1 mixture of **b** EcN *znuA zupT* and *znuA zupT irp2* (mice were 21 weeks of age) or **c** EcN *znuA zupT* and *znuA zupT ybtX* (mice were 30 weeks of age). Fecal samples were collected daily and the competitive index (C.I.) was calculated by dividing the output ratio (CFU of EcN *znuA zupT* / CFU of the respective EcN triple mutant) by the CFU-enumerated input ratio of the strains. Two-sided one-sample *t* test was used on log-transformed data to accept or reject null hypothesis (theoretical mean = 0). **d** mRNA expression of *S100a8*, *S100a9,* and *Lcn2* was measured in the cecum of mice in panel **c** (*n* = 5 biologically independent samples); conventional DSS-treated mice colonized with EcN were used as a control (*n* = 5 biologically independent samples). Each circle represents a biologically independent sample, and bars represent the geometric mean. Unpaired two-tailed *t* test was used. **e** Experiment timeline for panels **f** and **g**. **f**, **g** Male and female *S100a9*^-/-^ mice were given 4% (w/v) DSS in the drinking water for 4 days (day −4 to 0). On day 0, mice were orally gavaged with 1 × 10^9^ CFU of a 1:1 mixture of **f** EcN *znuA zupT* and *znuA zupT irp2* (mice were 9–10 weeks of age) or **g** EcN *znuA zupT* and *znuA zupT ybtX* (mice were 16–30 weeks of age). Fecal samples were collected daily and the C.I. was calculated as described for panel **b** strains. Two-sided one-sample *t* test was used on log-transformed data to accept or reject null hypothesis (theoretical mean = 0). **b**, **c**, **f**, **g** Each data point represents a single mouse (biologically independent samples), and bars represent the geometric mean. The number (=n) of biologically independent replicates for each group is indicated in each figure panel. **h** mRNA expression of *Lcn2, Il17a, Tnfa, and Ifng* was measured in the cecum of mice in panel **f**. Each circle (*n* = 5) represents a biologically independent sample, and bars represent the geometric mean. **i** Representative stained sections (H&E, original magnification ×10, scale bars represent 250 μm) of colon from germ-free mice, or DSS-treated *S100a9*^−/−^ mice, 5–7 days post-administration of different groups of EcN (germ-free EcN *znuA zupT* vs *znuA zupT irp2*, *n* = 6; germ-free EcN *znuA zupT* vs *znuA zupT ybtX*, *n* = 5; *S100a9*^−/−^ mice EcN *znuA zupT* vs *znuA zupT irp2*, *n* = 6; *S100a9*^−/−^ mice EcN *znuA zupT* vs *znuA zupT ybtX*, *n* = 5; all colon samples are biologically independent). *P value ≤ 0.05; **P value ≤ 0.01; ***P value ≤ 0.001, ns not significant. Exact *P* values are reported in Supplementary Data 2. Source data are provided as a Source Data file.

Next, we ascertained whether the zinc transport systems of EcN play a significant role in the absence of gut inflammation. As EcN colonization levels decline over time in conventional mice in the absence of inflammation, we used germ-free mice (Fig. 5a), in which we previously observed high levels of EcN colonization for extended periods of time^3^. When we inoculated germ-free mice with a 1:1 mixture of EcN *znuA zupT* and either *znuA zupT irp2* (Fig. 5b) or *znuA zupT ybtX* (Fig. 5c), we recovered similar amounts of each strain from mouse feces throughout the experiment (Fig. 5b, c and Supplementary Fig. 5a, b). Whereas *S100a8*, *S100a9*, and *Lcn2* were highly expressed in the ceca of DSS-treated animals colonized with EcN, these genes were only minimally upregulated (< 10-fold) in germ-free mice colonized with EcN (Fig. 5d). The absence of inflammation in EcN*-*colonized germ-free mice was also confirmed by colon pathology (Fig. 5i and Supplementary Fig. 5e).

To further probe whether Ybt provides a means for EcN to evade CP-dependent zinc depletion in vivo, we employed *S100a9*^*−/−*^ mice (deficient in CP) treated with DSS (Fig. 5e), and inoculated them with a 1:1 mixture of EcN *znuA zupT* and either *znuA zupT irp2* (Fig. 5f) or *znuA zupT ybtX* (Fig. 5g). We recovered similar amounts of each strain from these mice lacking CP (Fig. 5f, g and Supplementary Fig. 5c, d) even though the mice developed intestinal inflammation, as indicated by high expression levels of pro-inflammatory genes *Lcn2*, *Il17a*, *Tnfa*, and *Ifng* (Fig. 5h) and by analysis of colon pathology (Fig. 5i and Supplementary Fig. 5e). Our results thus indicate that Ybt confers a colonization advantage to EcN in the inflamed gut, by enabling EcN to evade CP-dependent zinc sequestration.

## Discussion

Commensal and pathogenic *Enterobacteriaceae* exploit host inflammation to achieve high levels of colonization and outcompete obligate anaerobes; these mechanisms include the ability to utilize alternative electron acceptors that become available following the production of reactive oxygen and nitrogen species by activated host cells^5,7^, as well as new nutrient sources such as lactate^55^ and acidic sugars^56^. In addition to taking advantage of new metabolic resources, *Enterobacteriaceae* must also overcome host-mediated mechanisms of nutritional immunity^13^, including metal ion starvation^57^.

We have previously shown that pathogenic STm and probiotic EcN evade lipocalin-2-mediated iron sequestration in the inflamed gut via the production of stealth siderophores^16,33^. As we have found that STm also evades CP-mediated zinc sequestration in the inflamed gut^15^, we sought to investigate whether EcN also evades CP to acquire zinc and thrive in the host. As EcN, akin to STm, expresses ZnuABC and ZupT, we initially hypothesized that these zinc transporters mediate EcN resistance to CP. However, when we found that an EcN *znuA zupT* mutant still grew up to 1000-fold better than an STm *znuA zupT* mutant in media containing CP (Fig. 1), we speculated that EcN must utilize additional mechanisms to acquire zinc. In the work presented herein, we unexpectedly discovered that EcN scavenges zinc with the siderophore Ybt.

Ybt is a phenolate siderophore that was first discovered as being produced by *Yersinia enterocolitica*^58^. The term siderophore has its origin in the Greek language and means “iron carrier”, as these molecules are widely characterized as being produced by microorganisms to acquire iron. However, recent studies have proposed that at least some siderophores may also bind to other metals. For example, the siderophore ferrioxamine was shown to bind manganese^59,60^, and Ybt was shown to bind copper as a means to evade toxicity^61^ and to scavenge copper and nickel in vitro^62,63^. Nevertheless, the extent and biological relevance for siderophores binding to other metals remains largely unknown.

Most of the genes involved in Ybt biosynthesis are grouped in a gene cluster^64,65^. In addition to *Yersinia* species, many *Enterobacteriaceae* also produce Ybt, including both pathogenic and commensal *E. coli*^46,66–68^. Ybt is well known for scavenging iron in vivo^69^, and plays a critical role in *Y. pestis* virulence^64^. Moreover, Ybt reduces reactive oxygen species formation in phagocytes by scavenging iron and preventing Haber-Weiss reactions^70^, as well as contributes to intestinal fibrosis^66^, indicating that Ybt modulates the host immune response. Incidentally, a product of the *ybt* gene cluster has been proposed to enable zinc acquisition by *Y. pestis*^40^, although direct binding of Ybt to zinc was not described in two independent studies^39,41^. Our finding that pH influences binding of Ybt to zinc is likely a key reason for the lack of binding that was observed in these prior publications, as they did not assess changing the pH. Moreover, reinterpretation of the original NMR and UV data in the aforementioned studies does suggest that at least partial zinc coordination can be seen, as the data show slight UV and NMR shifts that are consistent with only a small amount of Ybt being bound to zinc. Nevertheless, a critical question remained as to the identity of the molecule(s) produced by the Ybt gene cluster that contributed to zinc acquisition and, in the context of our study, whether such a molecule could play a role in gut colonization.

Using UHPLC-MS/MS, we identified two diastereomers of Ybt from EcN wild-type supernatant extract that were not present in the *irp2* mutant supernatant; MS/MS spectra of both peaks matched the MS/MS spectrum of commercial Ybt. Ybt is known to isomerize at the C10 position (Fig. 3) into a racemic mixture^49^. Using post-LC pH neutralization and metal infusion in a recently developed workflow termed native metabolomics, we found that one isomer (retention time = 4.0 min) preferentially binds zinc (Fig. 2). The different affinity of siderophore diastereomers for a metal is not unprecedented. Pyochelin, a siderophore with a similar thiazoline core as Ybt and produced by *Burkholderia cepacia* and several *Pseudomonas* strains, also exists as two diastereomers, only one of which binds iron^71^. Moreover, although pyochelin was shown to bind both iron and zinc in vitro^72^, to our knowledge, the biological relevance of pyochelin-mediated zinc scavenging has not been investigated. Similarly, only one of the Ybt isomers was shown to bind gallium when the compound’s structure was initially characterized^49^. We used 1D ^1^H NMR spectroscopy to confirm this observed zinc binding by Ybt (Fig. 3). Specifically, we observed the loss of signal corresponding to the two OH groups and the NH proton in Ybt, which indicates the corresponding heteroatoms that chelate the Zn^2+^ ion. Although the other zinc-coordinating atoms were not directly characterized, we hypothesize that Ybt binds zinc in the same manner as it binds iron and as micacocidin A binds Zn^2+^.

Because Ybt is known to bind iron, we performed a competition assay with equimolar amounts of zinc and iron. We observed that the metal-binding preference of Ybt is pH-dependent – Ybt preferentially binds to zinc in basic conditions (pH = 10), to iron in acidic conditions (pH = 4), and exhibits similar preference for both at pH 7 (Fig. 2e–g). In contrast to Ybt, the binding capacity of pyochelin to different metals is pH independent^72^. We speculate that the pH-dependent metal selectivity of Ybt may confer different functions to it under various physiological conditions (e.g., inflammation or homeostasis), or different colonization niches. For instance, in healthy human subjects, the pH in the small intestine gradually increases from pH 6 in the duodenum to about pH 7.4 in the terminal ileum, then drops to pH 5.7 in the caecum, but again gradually increases, reaching pH 6.7 in the rectum^73^. Upon inflammation, the pH in most sections of the gastrointestinal tract further decreases, but the colon still possesses a higher pH than the small intestine and cecum. Because dietary iron is mainly absorbed in the small intestine, the ability of Ybt to bind iron at lower pH may enable EcN and other Ybt-producing bacteria to compete with the host for iron in the small intestine. On the other hand, the zinc-binding ability of Ybt may enhance colonization of Ybt-producing bacteria in the colon, where the pH is higher. Intriguingly, in patients with active inflammatory bowel disease, the pH in many sections of the intestine increases; for example, the terminal ileum has been observed to reach up to pH 9.2^74^.

During colitis, neutrophils are recruited to sites of inflammation and secrete high levels of CP to sequester zinc from invading pathogens^15,25,75^. Our observation that Ybt renders EcN more resistant than STm to zinc sequestration by CP (Fig. 1) in vitro prompted us to investigate the function of Ybt during EcN colonization of the inflamed gut. We found that EcN mutants lacking either Ybt or the putative inner membrane receptor YbtX, in addition to lacking ZnuABC and ZupT, showed more severe colonization defects than the *znuA zupT* mutant in mice with DSS-induced colitis (Fig. 4). As four other iron transport systems (including the stealth siderophores salmochelin and aerobactin, as well as heme uptake) are still present in these strains, it is unlikely that the in vivo phenotype of the mutants is due to an inability to overcome iron starvation. Consistent with this hypothesis, the growth defect of the triple mutants (*znuA zupT irp2* and *znuA zupT ybtX*) in minimal media was only rescued by zinc supplementation, but not by iron or nickel supplementation (Fig. 1). Future studies with strains lacking Ybt in combination with other iron or nickel acquisition systems are necessary to define the contribution of Ybt for acquisition of these metals in vivo.

Together with the observations that Ybt contributes to optimal growth of EcN in zinc-limited conditions in vitro (Fig. 1), and that Ybt directly binds zinc at the pHs found in the intestine (Fig. 2), the colonization defect of EcN *znuA zupT irp2* and of EcN *znuA zupT ybtX* in DSS-treated mice is consistent with the strains’ limited ability to acquire zinc. Moreover, the colonization advantage provided by Ybt is highly dependent on the state of inflammation and presence of CP, as EcN *znuA zupT* and EcN *znuA zupT ybtX* colonized to similar levels in *S100a9*^*−/−*^ mice as well as in germ-free mice (which lack inflammation and only express low levels of CP) (Fig. 5). These results are in agreement with the in vitro results showing that Ybt and the putative inner membrane permease YbtX enable EcN to acquire zinc in media supplemented with CP (Fig. 1). Our results are also in agreement with prior work in *Y. pestis* showing that YbtX is involved in zinc transport^40^.

Altogether, our work demonstrates that Ybt directly binds to zinc in a pH-dependent manner, and that EcN can use Ybt to scavenge zinc in physiologic, zinc-limiting in vitro conditions and in the inflamed gut, thus overcoming CP-mediated zinc limitation. Broadly, our study proposes that the role of Ybt and other siderophores may be more complex than previously thought and may involve scavenging zinc in the host. Because many commensal and pathogenic *Enterobacteriaceae* (including *Yersinia* spp., *E. coli*, and *Klebsiella pneumoniae*) produce Ybt, this important mechanism of zinc acquisition in the gut may also play a role in other host tissues where pathogens must scavenge zinc.

## Methods

### Bacterial strains, plasmids, and growth conditions

Bacterial strains and plasmids are listed in Supplementary Table 1. Cultures of STm and *E. coli* were routinely incubated either aerobically at 37 °C in Lysogeny broth (LB; per liter: 10 g tryptone, 5 g yeast extract, 10 g NaCl) or on LB agar plates (1.5% Difco agar) overnight. Antibiotics and other chemicals were added at the following concentrations (mg/L) as needed: carbenicillin (Carb), 100; chloramphenicol (Cm), 30; kanamycin (Km), 50 or 100; nalidixic acid (Nal), 50; and 5-bromo-4-choloro-3-indoyl-ß-d-galactopyranoside (Xgal), 40. For counterselection of pJK611, 10 % (w/v) sucrose was added to media.

### *E*. *coli* Nissle 1917 mutant generation

Mutants in EcN and STm were constructed using either the lambda Red recombinase system or allelic exchange deletion. To generate mutants with the lambda Red recombinase system^76^, primers (Supplementary Table 2) homologous to sequences flanking the 5′ and 3′ ends of the target regions were designed and were used to replace the selected genes with a chloramphenicol (derived from pKD3), a kanamycin (derived from pKD4), or a tetracycline resistance cassette (Supplementary Table 2). Strain names for the mutants are listed in Supplemental Table 1. To confirm integration of the resistance cassette and deletion of the target, mutant strains and wild-type controls were each assayed utilizing PCR, and sequencing primers (Supplementary Table 2) that flank the target sequence were used in conjunction with a common test primer to test for both new junction fragments. *E. coli* Nissle 1917 wild type as well as mutants *znuA zupT*, *znuA zupT ybtX*, and *znuA zupT irp2* were sequenced to confirm strain identity and to identify any unexpected mutations. Quality-control metrics for next-generation sequencing data were obtained using FastQC. Raw reads were aligned to the *E. coli* Nissle 1917 reference genome (Genbank accession CP022686.1) using BWA MEM^77^. PCR duplicates were removed using Picard MarkDuplicates^78^ to ensure that downstream results are not biased by PCR duplication artifacts. Variants were discovered using freeBayes^79^, and Annovar^80^ was used to annotate variants with overlapping gene annotation information and effect on gene and protein sequence, predicted effect on protein structure and function, and previous observation of that variant in refGene database (Supplementary Data 1).

### Allelic exchange deletion of *znuA* in *E. coli* Nissle 1917

To construct an *E. coli* Nissle 1917 (EcN; Supplementary Table 1) *znuA* mutant, DNA regions of ~600–800 bp in length flanking the *znuA* gene were amplified by PCR with primers *znuA* FR1-Fw and *znuA* FR1-Rv (upstream flanking region, FR1) and primers *znuA* FR2-Fw and *znuA* FR2-Rv (downstream flanking region, FR2). A BamHI restriction site was added to the 5′ end of primers *znuA* FR1-Fw and *znuA* FR2-Rv, and an XbaI restriction site was added to the 5′ end of primers *znuA* FR1-Rv and *znuA* FR2-Fw (Supplementary Table 2). The blunt-end flanking region PCR products were digested with XbaI, then ligated together using the Quick Ligation kit (New England Biolabs). Phusion High Fidelity DNA polymerase and primers *znuA* FR1-Fw and *znuA* FR2-Rv were used to amplify the FR1 + FR2 ligation product (FR1-FR2). A PCR product of the predicted size was gel-purified and ligated into pCRBlunt II-TOPO using the ZERO Blunt cloning kit (Invitrogen). The construct was heat-shocked into *E. coli* TOP10 and plated on LB + Kan plates. Plasmids isolated from single colonies were screened by EcoRI digestion, and positive clones were confirmed by sequencing using M13 Fw and M13 Rv universal primers. Accurate clones were designated pNM3 (pCRBlunt II-TOPO::*znuA* FR1-FR2). The plasmid pNM3 was digested with BamHI, and the FR1-FR2 fragment was gel-purified, ligated to BamHI-digested suicide vector pRDH10, then introduced into chemically competent *E. coli* CC118 λpir cells by heat-shock followed by selection on LB + Cm agar. A positive clone was designated pNM4 (pRDH10::*znuA* FR1-FR2). To enable conjugation of pNM4, purified plasmid was heat-shocked into *E. coli* S17-1 λpir. Wild-type EcN carrying the temperature-sensitive plasmid pSW172^81^ was conjugated with *E. coli* S17-1 λpir containing pNM4 on LB agar at 30 °C (to allow for pSW172 replication). Single-crossover transconjugants were then selected for on LB + Carb + Cm agar. Afterwards, the transconjugants were subjected to sucrose selection in order to counterselect cells still harboring the integrated pNM4 plasmid, thus yielding WT revertants or Δ*znuA* mutants. Mutants were confirmed by PCR, then were cured of pSW172 by growth at 37 °C. The resulting mutant strain was termed JZL95 (EcN Δ*znuA*(−82 to +1000)). An EcN Δ*znuA*::KSAC strain was also generated (JZL109), first cloning the XbaI-digested KSAC kanamycin resistance cassette from pBS34 into the XbaI restriction site of pNM4, yielding pNM4::KSAC (pRDH10::*znuA* FR1-KSAC-FR2).

### Construction of EcN *znuA zupT* mutant

The EcN *znuA zupT* mutant was constructed using the lambda Red recombinase system. Briefly, primers (*zupT*-Fw and *zupT*-Rv) were designed with sequences homologous to the 5′ and 3′ ends of the EcN *zupT* gene and sequences homologous to the kanamycin resistance cassette of pKD4. The primers were used to PCR amplify the kanamycin resistance cassette. The PCR product was gel-purified, then electroporated into EcN *znuA* (JZL95) carrying pJK611 (a sucrose-counterselectable variant of pKD46). Kanamycin and sucrose were used for selection of deletion mutants and counterselection of pJK611, respectively. Putative mutants were screened on LB + Carb agar to confirm loss of pJK611, and the mutation was then confirmed using PCR. The resulting strain was termed JZL100 (EcN Δ*znuA*(−82 to +1000) Δ*zupT*(−42 to +774)::Kan).

### Construction of EcN *ybtX, znuA ybtX*, and *znuA zupT ybtX* mutants

EcN *znuA ybtX* and EcN *znuA zupT ybtX* were constructed by allelic exchange, employing EcN *znuA* (JZL109) and EcN *znuA zupT* (JZL100) as parental strains. Using the NEBuilder tool (http://nebuilder.neb.com), overlapping primers were designed to construct a deletion vector for the *ybtX* gene (−108 to +1281). In all, 1000 bp upstream and downstream of the EcN *ybtX* gene, as well as the chloramphenicol resistance cassette from pKD3, were amplified with the specific primers. Suicide plasmid pGP704 was digested with EcoRV and SalI. PCR products and plasmids were gel-purified, then equimolar concentrations were used in a Gibson Assembly reaction, then transformed into *E. coli* CC118 λpir, selecting on LB + Cm agar. Clones were screened by sequencing the plasmid insert, and an accurate clone was designated pJB10 (pGP704::*ybtX* FR1-Cm-FR2). pJB10 was then electroporated into *E. coli* S17-1 λpir. EcN *znuA* and EcN *znuA zupT* were then separately conjugated with *E. coli* S17-1 λpir pJB10 on LB agar, after which transconjugants were selected for by plating on LB + Kan+Cm agar. Double-crossover mutants were identified by patching colonies on LB + Carb and LB + Cm plates. Carb^S^ Cm^R^ colonies were checked by PCR to assess for the loss of *ybtX* (ybtX_pres_Fw and ybtX_pres_Rv) and the presence of the chloramphenicol resistance cassette (Gib_out_LB_Fw and C2; Gib_out_RB_Rv and C1). The resulting strain EcN *znuA zupT ybtX* was termed JB76, while EcN *znuA ybtX* was termed JB92. The EcN *ybtX* mutant was constructed using the lambda Red system. Briefly, primers (ybtX_red_Fw and ybtX_red_Rv2) were designed with sequences homologous to the 5′ and 3′ ends of the EcN *ybtX* gene and to the chloramphenicol resistance cassette of pKD3. The primers were used to amplify the chloramphenicol resistance cassette by PCR. The PCR product was electroporated into EcN wild-type carrying pJK611. Chloramphenicol and sucrose were used for selection of deletion mutants and counterselection of pJK611, respectively. The mutation was confirmed using PCR, and pJK611 was confirmed to be cured by plating on LB + Carb agar. The resulting strain was termed JB90 (EcN Δ*ybtX*(−108 to +1281)::Cm).

### Construction of EcN *irp2* and EcN *znuA zupT irp2* mutants

A mutant in EcN carrying a deletion of the *irp2* open reading frame was constructed using the lambda Red system. Briefly, primers (irp2up-TetRA F and irp2dn-TetRA R) were designed with sequences homologous to the 5′ and 3′ ends of the EcN *irp2* gene and to the *tetRA* resistance cassette of MSC74. The primers were used to amplify the tetracycline resistance cassette by PCR. The PCR product was electroporated into wild-type EcN containing pJK611. Tetracycline and sucrose were used for selection of mutants and counterselection of pJK611, respectively. The *irp2* deletion was confirmed using PCR (irp2inF and irp2inR) and sequencing (irp2-upF and irp2-dnR), and also confirmed for the loss of plasmid pJK611 by plating on LB + Carb plates. The resulting strain was termed HZE116 (EcN Δ*irp2*::Tet). A deletion of the *irp2* open reading frame in EcN *znuA zupT* (JZL100) was constructed by allelic exchange. Using the NEBuilder tool (http://nebuilder.neb.com), overlapping primers were designed to construct a deletion vector for the *irp2* gene. 500 bp upstream (pGP704-salI US irp2 F and TetR-US irp2 R) and downstream (TetA-DS irp2 F and pGP704-sacI DS-irp2 R) regions of EcN flanking the *irp2* gene, as well as the tetracycline resistance cassette (TetR-F and TetA-R) of MSC74, were then amplified. Suicide plasmid pGP704 was digested with SalI and SacI. PCR products and plasmid were gel-purified, then equimolar concentrations were used in a Gibson Assembly reaction, followed by transformation into DH5α λ*pir* and selection on LB + Tet agar. Clones were screened by sequencing, and an accurate clone was designated pHZE107 (pGP704::*ybtX* FR1-Tet-FR2). pHZE107 was then purified and electroporated into *E. coli* S17-1 λpir. EcN *znuA zupT* was conjugated with *E. coli* S17-1 λpir pHZE107 on LB agar. Transconjugants were then selected for by plating on LB + Kan+Tet, and double-crossovers were identified by screening for Carb^S^ colonies. Kan^R^ Tet^R^ Carb^S^ colonies were then tested by PCR with primers checking for the loss of *irp2* (irp2inF and irp2inR) and the presence of the tetracycline resistance cassette (TetR-F and TetA-R). The resulting strain was termed HZE112 (EcN *znuA zupT irp2*).

### Bacterial Growth in LB, modified LB supplemented with calprotectin, and M9 minimal medium

STm and EcN strains were tested for their ability to grow in nutrient-rich conditions (LB), nutrient-limited conditions (M9 minimal medium per liter; 6.8 g Na_2_HPO_4_, 3 g KH_2_PO_4_, 0.5 g NaCl, 1 g NH_4_Cl, 0.1 mM CaCl_2_, 1 mM MgSO_4_, 0.2% glucose), and in modified LB supplemented with calprotectin (CP). Bacteria were inoculated into M9 minimal medium from an LB agar plate, then shaken overnight at 37 °C. Absorbance (*λ* = 600 nm) of the overnight cultures was determined by spectrophotometry, 10^9^ colony-forming units (CFU) were harvested by centrifugation, washed with M9 medium twice, then serially diluted in M9. For Fig. 1e, f, M9 additionally contained 1 mg/ml biotin and 1 mg/ml thiamin and strains were grown in LB instead of M9 overnight. For growth assays, 5 µl of the 10^7^ CFU/ml dilution were used to inoculate 95 μl of LB or M9. Cultures were incubated for 24 h at 37 °C with shaking and growth was assessed at different time points by CFU enumeration on agar plates. Growth was also tested in M9 minimal medium supplemented with 5 μM ZnSO_4_, 5 μM FeCl_2_, or 5 μM NiCl_2_. 1 μM apo-yersiniabactin (EMC Microcollections), or 1 μM apo-enterobactin (kindly provided by Dr. Elizabeth Nolan, MIT). For growth in modified LB supplemented with CP, 10 µl of 10^5^ CFU/ml was used to inoculate 90 μl of LB supplemented with CP buffer (20 mM Tris pH 7.5, 100 mM β-mercaptoethanol, 3 mM CaCl_2_) and 125, 150 or 250 μg/ml wild-type CP, or 150 μg/ml Site I/II mutant CP (MU CP), respectively (10:28:62 ratio of inoculum to LB media to CP buffer). CP was produced as described previously^37^, and two batches were used for this study. Growth was assessed by enumerating CFU on agar plates after incubating cultures statically for 16 h at 37 °C.

### Yersiniabactin standard sample preparation for MS

Yersiniabactin (acquired from EMC Microcollections, https://www.microcollections.de/) stock solutions were prepared by resuspension of the compound in ethanol to a concentration of 1 mM. A 20 µM solution was prepared for mass spectrometry (MS) analysis. Final solutions for analysis were prepared in 50% methanol/50% water or in water + 0.1% formic acid (pH 2.8).

### *E. coli* Nissle sample preparation for MS

Supernatants from wild-type and *irp2* knockout *E. coli* Nissle cultures were extracted onto pre-washed SPE cartridges. SPE cartridges were activated 3x with MeOH (3 × 3 ml), then were washed 2x with water + 0.1% formic acid (3 × 3 ml). Sample was loaded dropwise (steady single dripping) onto SPE cartridges, then cartridges were washed with water + 0.1% formic acid (3 × 3 ml). Sample was eluted into 1.9 ml MeOH, then this was concentrated by speed evaporation at room temperature. Samples were weighed and reconstituted with 80% MeOH/20% water + 0.1% FA to a final concentration of 1 mg/ml. 5 µl of sample were injected per run.

### Direct Inject-MS data acquisition

For MS analysis, 5 µl were injected through a Vanquish UHPLC system into a Q Exactive Orbitrap mass spectrometer (Thermo Fisher Scientific, Bremen, Germany). A flow rate between 0.2 ml/min and 0.4 ml/min was used for experiments. Data acquisition was performed in MS1 in positive mode. Electrospray ionization (ESI) parameters were set to 52 L/min sheath gas flow, 14 L/min auxiliary gas flow, 0 L/min sweep gas flow, and 400 °C auxiliary gas temperature. The spray voltage was set to 3.5 kV and the inlet capillary to 320 °C. 50 V S-lens level was applied. MS scan range was set to 150–1500 m/z with a resolution at m/z 200 (*R*_m/z 200_) of 35,000 with one micro-scan. The maximum ion injection time was set to 100 ms with an automated gain control (AGC) target of 1.0E6.

### UHPLC-MS/MS data acquisition

For LC-MS/MS analysis, 5 µl were injected into a Vanquish UHPLC system coupled to a Q Exactive Orbitrap mass spectrometer (Thermo Fisher Scientific, Bremen, Germany). For the chromatographic separation, a C18 porous core column (Kinetex C18, 50 × 2 mm, 1.8 μm particle size, 100 Angstrom pore size, Phenomenex, Torrance, USA) was used. For gradient elution, a high-pressure binary gradient system was used. The mobile phase consisted of solvent A (H_2_O + 0.1% FA) and solvent B (acetonitrile + 0.1% FA). The flow rate was set to 0.5 ml/min. After injection, the samples were eluted with the following linear gradient: 0–0.5 min 5% B, 0.5–5 min 5–99% B, followed by a 2 min washout phase at 99% B and a 3 min re-equilibration phase at 5% B. Data-dependent acquisition (DDA) of MS/MS spectra was performed in positive mode. ESI parameters were set to 52 L/min sheath gas flow, 14 L/min auxiliary gas flow, 0 L/min sweep gas flow, and 400 °C auxiliary gas temperature. The spray voltage was set to 3.5 kV and the inlet capillary to 320 °C. 50 V S-lens level was applied. MS scan range was set to 150–1500 m/z with a resolution at m/z 200 (R_m/z 200_) of 35,000 with one micro-scan. The maximum ion injection time was set to 100 ms with an AGC target of 1.0E6. Up to 5 MS/MS spectra per MS1 survey scan were recorded in DDA mode with R_m/z 200_ of 17,500 with one micro-scan. The maximum ion injection time for MS/MS scans was set to 100 ms with an AGC target of 3.0E5 ions and minimum 5% C-trap filling. The MS/MS precursor isolation window was set to m/z 1. Normalized collision energy was set to a stepwise increase from 20 to 30 to 40% with *z* = 1 as default charge state. MS/MS scans were triggered at the apex of chromatographic peaks within 2 to 15 s from their first occurrence. Dynamic precursor exclusion was set to 5 s. Ions with unassigned charge states were excluded from MS/MS acquisition as well as isotope peaks.

### Post LC-MS/MS pH neutralization and metal addition for native spray mass spectrometry

A stock solution of 160 mM Zn(CH_3_CO_2_)_2_ was prepared, then diluted to a final concentration of 3.2 mM. A stock solution of ammonium hydroxide at 1 M was also prepared. Sample was run through a C18 column at a flow rate of 0.5 ml/min. Before electrospray, a neutralizing solution of 1 M ammonium hydroxide was added at a flow rate of 5 μl/min, then the solution of 3.2 mM zinc acetate was added at a flow rate of 5 μl/min. Post-LC pH was verified by collecting the flow through and spotting on pH paper (Sigma).

### Ion identity molecular networking of wild-type *E. coli* Nissle supernatant extracts with post-LC zinc infusion

MS was run as described in the LC-MS/MS data acquisition section. MS/MS spectra were converted to.mzML files using MSconvert (ProteoWizard)^82^. All raw and processed data is publicly available at https://massive.ucsd.edu/ProteoSAFe/dataset.jsp?task=cf9f1c6478964a6f9464006b7aa9bd49. MS1 feature extraction and MS/MS pairing was performed with MZMine 2.37corr17.7_kai_merge2^34,83,84^. An intensity threshold of 1E6 for MS1 spectra and of 1E3 for MS/MS spectra was used. MS1 chromatogram building was performed within a 10 ppm mass window and a minimum peak intensity of 3E5 was set. Extracted Ion Chromatograms (XICs) were deconvoluted using the local minimum search algorithm with a chromatographic threshold of 0.01%, a search minimum in RT range of 0.1 min, and a median m/z center calculation with m/z range for MS2 pairing of 0.01 and RT range for MS2 scan pairing of 0.2. After chromatographic deconvolution, MS1 features linked to MS/MS spectra within 0.01 m/z mass and 0.2 min retention time windows. Isotope peaks were grouped and features from different samples were aligned with 10 ppm mass tolerance and 0.1 min retention time tolerance. MS1 peak lists were joined using an m/z tolerance of 10 ppm and retention time tolerance of 0.1 min; alignment was performed by placing a weight of 75 on m/z and 25 on retention time. Gap filling was performed using an intensity tolerance of 10%, an m/z tolerance of 10 ppm, and a retention tolerance of 0.1. Correlation of co-eluting features was performed with the metaCorrelate module; retention time tolerance of 0.1, minimum height of 1E5, noise level of 1E4 were used. A correlation of 85 was set as the cutoff for the min feature shape corr. The following adducts were searched: [M + H^+^]^+^, [M + Na^+^]^+^, [M + K^+^]^+^, [M + Ca^2+^]^2+^, [M + Zn^2+^- H^+^]^+^, and [M-H2O], with an m/z tolerance of 10 ppm, a maximum charge of 2, and maximum molecules/cluster of 2. Peak areas and feature correlation pairs were exported as.csv files and the corresponding consensus MS/MS spectra were exported as an.mgf file. For spectral networking and spectrum library matching, the.mgf file was uploaded to the feature-based molecular networking workflow on GNPS (gnps.ucsd.edu)^47,48,85^. For spectrum library matching and spectral networking, the minimum cosine score to define spectral similarity was set to 0.7. The Precursor and Fragment Ion Mass Tolerances were set to 0.01 Da and Minimum Matched Fragment Ions to 4, Minimum Cluster Size to 1 (MS Cluster off). When Analog Search was performed, the maximum mass difference was set to 100 Da. The GNPS job for the siderophore mix can be accessed: https://gnps.ucsd.edu/ProteoSAFe/status.jsp?task=525fd9b6a9f24455a589f2371b1d9540. All.csv and.mgf files in addition to MZmine 2 project can be accessed at https://massive.ucsd.edu/ProteoSAFe/dataset.jsp?task=cf9f1c6478964a6f9464006b7aa9bd49. Mgf files were exported for SIRIUS in MZmine2, then molecular formulas were determined using SIRIUS 4.0.1 (build 9)^86^ and molecular formulas can be accessed: http://gnps.ucsd.edu/ProteoSAFe/status.jsp?task=e2bd16458ec34f3f9f99982dedc7d158.

### Metal competition MS experiments

Commercial yersiniabactin (dissolved to 1 mM in ethanol) was added to 10 mM ammonium acetate buffer at a defined pH (as determined by pH meter, Denver Instrument UltraBasic) for a final concentration of 10 µM. Acetic acid was added to the buffer to lower the pH to 4, and ammonium hydroxide was added to the buffer to raise the pH to 10. Solutions of zinc acetate and iron chloride were prepared to a final concentration of 10 mM in water; from this solution, both iron and zinc were added to a final solution of 100 µM.

### Yersiniabactin/calprotectin competition assay

2 mg of calprotectin and mutant-calprotectin in 200 μL buffer A (20 mM Tris pH 7.5, 100 mM β-mercaptoethanol, 3 mM CaCl2) were exchanged into 10 mM ammonium acetate buffer using Amicon Ultra 3k filters (14,000×*g* for 15 mins, 3x). Calprotectin and mutant-calprotectin were reconstituted in a 10 mM ammonium acetate buffer to a final concentration of 4 mg/mL. Then calprotectin and mutant-calprotectin were incubated with 168 μM ZnSO_4_ (or water vehicle) for 90 mins at a 1:1 ratio (calprotectin contains 2 zinc-binding sites). Yersiniabactin was incubated with 100 μM ZnSO_4_ (or water vehicle) then was added to calprotectin (or mutant-calprotectin) at a final ratio of 2:1 protein to yersiniabactin. This was incubated overnight at 4 °C, then yersiniabactin was separated from protein using Amicon Ultra 3k filters (14,000×*g* for 30 mins) by collecting the flowthrough. Flowthrough was run using direct infusion mass spectrometry in technical duplicate. For MS analysis, 5 µL of flowthrough were directly infused into a Q-Exactive orbitrap mass spectrometer via flow injections through a Vanquish UHPLC system (Thermo Fisher Scientific, Bremen, Germany). A flow rate of 0.15 mL/min was used. MS1 data acquisition was performed in positive mode. Electrospray ionization (ESI) parameters were set to 53 L/min sheath gas flow, 14 L/min auxiliary gas flow, 0 L/min sweep gas flow, and 400 °C auxiliary gas temperature. The spray voltage was set to 3.5 kV and the inlet capillary to 320 °C. 50 V S-lens level was applied. MS scan range was set to 200–2000 m/z with a resolution at m/z 200 (Rm/z 200) of 70,000 with one micro-scan. The maximum ion injection time was set to 200 ms with an automated gain control (AGC) target of 3.0E6. Peak area was integrated using Quant Browser in Thermo XCalibur software, and data was visualized using GraphPad Prism 9.

### NMR experiments and signal assignments

All NMR experiments were performed on a Varian 500 MHz spectrometer equipped with a ^1^H channel cold-probe. The yersiniabactin (Ybt, 0.25 mg) sample was dissolved in deuterated acetonitrile (CD_3_CN, 300 µl). The NMR spectra were acquired at 298 K in 3-mm NMR tubes and raw data were processed using Bruker Topspin version 4.0.7. The ^1^H peaks resonance assignments were made using a combination of 2D COSY (Supplementary Fig. 2b), 2D ROESY with a mixing time of 300 ms (Supplementary Fig. 2c). The assignments were made with the assistance of NMRFAM-SPARKY. The 1D experiments were acquired with a relaxation delay of 1 s, 90° ^1^H pulses of about 9.0 µs, and a spectral width of 8000 Hz. 2D ROESY spectra were acquired with a spinlock of 200–300 ms, using 128 transients per FID, and 128 points in the indirect dimension.

### Zn binding and base titration for NMR studies

1D ^1^H NMR titration experiments were carried out to investigate the binding of Zn^2+^ (added as ZnCl_2_) to Ybt. Ybt (0.25 mg) was dissolved in deuterated acetonitrile (CD_3_CN, 300 μl) then a baseline spectrum was taken. A spectrum was recorded between each addition of ZnCl_2_. 0.5 equiv. of ZnCl_2_ dissolved in CD_3_CN was added to the dissolved Ybt, followed by a second 0.5 equiv. (1 equiv. total), another 1 equiv. (2 equiv. total), and finally another 3 equiv. (5 equiv. total). Once the spectrum with 5 equiv. of ZnCl_2_ was recorded, an NaOD (in D_2_O) titration was performed to determine the effect of increasing pH on the binding of Zn^2+^. Sequential additions of 0.5 equiv., a second 0.5 equiv., 1 equiv., and 3 equiv. were made and 1D ^1^H NMR spectra were recorded for each titration point.

### Mouse experiments

Germ-free Swiss Webster mice as well as specific pathogen-free C57BL/6 wild-type mice and *S100a9*^−/−^ mice were used in our study, in accordance with protocols and guidelines approved by the Institutional Animal Care and Use Committee of the University of California, Irvine (2009-2885; 2009-2872; 2015-3159) and the University of California, San Diego (S17107), and the University of Illinois, Chicago (20-016). C57BL/6 mice were purchased from the Jackson Laboratory, whereas *S100a9*^−/−^ mice^87^ were bred in-house. Mice at UC Irvine and at UC San Diego were fed diet Teklad 2920X. Germ-free Swiss Webster mice were purchased from Taconic Farms and then bred in-house in germ-free isolators (Park Bio). These mice were fed irradiated diet Purina 5066. The mice were kept in a 12 h light/dark cycle, at a room temperature of ~22 °C and ~52% humidity. For experiments, germ-free mice were transferred to sterile housing inside a biosafety cabinet, then colonized with the respective bacterial strains. For chemical colitis experiments using dextran sodium sulfate (DSS), mice were administered 4% (w/v) DSS (MP Biomedicals) in the drinking water beginning 4 days prior to administering bacteria, then provided a fresh 4% DSS solution one day prior. On the day of inoculation, mice were switched to 2% (w/v) DSS in the drinking water and orally gavaged with 1×10^9^ CFU of a mixture of strains at a 1:1 ratio, as indicated. A fresh 2% DSS solution was provided on day 4 post-inoculation. At day 5 or 7 post-inoculation, depending on weight loss, mice were humanely euthanized. Fecal content was collected on days 1, 4, and 5 or 7, whereas cecal content was collected directly during necropsy at day 7. CFU were enumerated by plating on appropriate selective agar media. In all mixed inoculation experiments, the competitive index of the EcN strains used in each group were calculated. Groups of 5–10 male and female mice were used for each experiment.

### Quantitative real-time PCR

Total RNA was extracted from cecal and colon tissues of wild-type mice, or cecal tissues of germ-free mice, with TRI Reagent (Sigma-Aldrich), followed by processing with an RNeasy Mini Plus kit (Qiagen). For analyzing gene expression by quantitative real-time PCR, cDNA from each RNA sample was prepared with the SuperScript IV VILO Master Mix with ezDNase kit (ThermoFisher). Real-time qPCR was performed with PowerUp SYBR Green Master Mix (ThermoFisher) and a QuantStudio 5 (ThermoFisher). Data were analyzed using the comparative 2^−ΔΔCt^ method. Target gene expression in each tissue sample was normalized to the respective levels of *Actb* mRNA (β-actin), and compared to uninfected samples.

### Histopathology

Distal colonic tissues from wild-type mice, proximal colonic tissues from *S100a9*^*−/−*^ mice, and proximal colonic tissues from germ-free mice were fixed in 10% buffered formalin, then processed according to standard procedures for paraffin embedding. 5 µm sections were stained with hematoxylin and eosin, then slides were scanned on a NanoZoomer Slide scanner (Hamamatsu) and scored in a blinded fashion as previously described^3^, with minor modifications. Briefly, each of four histological criteria (mononuclear infiltration, edema, epithelial injury, and neutrophilic inflammation/crypt abscesses) was determined as absent (0), mild (1), moderate (2), or severe (3). Furthermore, each parameter was assigned an extent factor reflecting its overall involvement ranging from 1 (<10%), 2 (10–25%), 3 (25–50%), and 4 (>50%). Scores represent the sum of the above scores in colon sections.

### Statistics

Statistical analysis was performed with GraphPad Prism 9. CFU data were transformed to Log_10_ and passed a normal distribution test before running statistical analyses. CFU from in vitro growth experiments were compared by one-way ANOVA (Fig. 1c, g) or two-way ANOVA (Fig. 1d) followed by Tukey’s multiple comparisons test, two-way ANOVA with Šidák’s multiple comparisons test (Fig. 1a, b), or one-sample *t* test (Supplementary Fig. 1a–e). Mouse experiments were analyzed with one-sample *t* test. Values were transformed to Log_10_ and compared to a hypothetical mean of 0. For comparison between groups, one-way ANOVA followed by Dunnet’s multiple comparisons test was used. An adjusted *P* value ≤ 0.05 was considered statistically significant. *P value ≤ 0.05, **P value ≤ 0.01, ***P value ≤ 0.001, ****P value ≤ 0.0001. For qPCR data analysis, a multiple *t*-test was performed on gene expression levels between the groups*;* * indicates a *P* value ≤ 0.05, **P value ≤ 0.01. Exact *P* values are reported in Supplementary Data 2.

### Reporting summary

Further information on research design is available in the Nature Research Reporting Summary linked to this article.

## Supplementary information

Supplementary Information

Description of Additional Supplementary Files

Supplementary Data 1

Supplementary Data 2

Reporting Summary

## Data Availability

Source Data for figures and all NMR raw data are provided with this paper. For genome analysis, we used the *E. coli* Nissle 1917 wild-type strain reference genome (GenBank CP022686.1). All mass spectrometry.raw and centroid.mzXML or.mzML files, in addition to MZmine 2 outputs and project file, are publicly available in the mass spectrometry interactive virtual environment (MassIVE) under massive.ucsd.edu with project identifier MSV000083387 (*E. coli* Nissle siderophores); raw spectra of yersiniabactin commercial standards are available under MSV000084237 (Siderophore Standard Mixture with metal additions). Ion Identity Molecular Networks can be accessed through gnps.ucsd.edu under direct links: https://gnps.ucsd.edu/ProteoSAFe/status.jsp?task=525fd9b6a9f24455a589f2371b1d9540 and http://gnps.ucsd.edu/ProteoSAFe/status.jsp?task=e2bd16458ec34f3f9f99982dedc7d158. Source data are provided with this paper.

## Source data

Source Data
