# Supplementary material for: Siderophore-mediated zinc acquisition enhances enterobacterial colonization of the inflamed gut
Source: Nat Commun. 2021 Dec 1;12:7016. doi: 10.1038/s41467-021-27297-2 (PMC8636617; doi:10.1038/s41467-021-27297-2)
Supplement: Supplementary file 1 — Supplementary Information [file 41467_2021_27297_MOESM1_ESM.pdf]

SUPPLEMENTARY INFORMATION

TABLES

Supplementary Table 1

**Strain or plasmid**	**Relevant characteristics**	**Source or Reference**
Escherichia coli strains		
CC118 λ*pir*	F- *araD139* Δ(*ara, leu*)7697 Δ*lacX74* *phoA*Δ20 *galE galK thi rpsE rpoB* $argE_{am}$ *recA1* λ*pir*	(Herrero et al., 1990)
DH5α λ*pir*	F- *supE44* Δ*lacU169* ϕ80d*lacZ*ΔM15 *recA1 endA1 hsdR17 thi-1 gyrA96 relA1* λ*pir*	(Platt et al., 2000)
DH5α MCR	F- *mcrA* Δ(*mrr-hsdRMS-mcrBC*) ϕ80d*lacZ*ΔM15 Δ(*lacZYA-argF)*U169 *deoR recA1 endA1 phoA supE44* λ- *thi-1 gyrA96 relA1*	(Grant et al., 1990)
S17-1 λ*pir*	F- *recA thi pro* r_K^- m_K^+ RP4::2-Tc::*Mu*Km Tn7 λ*pir*	(Herrero et al., 1990)
TOP10	F- *mcrA* Δ(*mrr-hsdRMS-mcrBC*) ϕ80d*lacZ*ΔM15 Δ*lacX74 recA1 araD139* Δ(*ara-leu*)7697 *galU galK rpsL*(Str^R) *endA1 nupG* λ-	Invitrogen
EcN	*E. coli* Nissle 1917 wild-type	Ardeypharm, Germany
JZL95 (*znuA*)	EcN Δ*znuA*(-82 to +1000)	This study
JZL109 (*znuA*)	EcN Δ*znuA*(-82 to +1000)::Kan	This study
JZL100 (*znuA zupT*)	EcN Δ*znuA*(-82 to +1000) Δ*zupT*(-42 to +774)::Kan	This study
JB90 (*ybtX*)	EcN Δ*ybtX*(-108 to +1281)::Cm	This study
JB92 (*znuA ybtX*)	EcN Δ*znuA*(-82 to +1000)::Kan Δ*ybtX*(-108 to +1281)::Cm	This study
JB76 (*znuA zupT ybtX*)	EcN Δ*znuA*(-82 to +1000) Δ*zupT*(-42 to +774)::Kan Δ*ybtX*(-108 to +1281)::Cm	This study
HZE116 (*irp2*)	EcN Δ*irp2*(+1 to +6108)::Tet	This study
HZE112 (*znuA zupT irp2*)	EcN Δ*znuA*(-82 to +1000) Δ*zupT*(-42 to +774)::Kan Δ*irp2*(+1 to +6108)::Tet	This study
Salmonella enterica serovar Typhimurium strains		

IR715	ATCC 14028s Nal^R	(Stojiljkovic et al., 1995)
JZL3	IR715 Δ*znuA*::Cm	(Liu et al., 2012)
APJ4	IR715 Δ*znuA*::Cm Δ*zupT*::KSAC	(Cerasi et al., 2013)
MSC74	IR715 Δ*iroN*::*tetRA*	(Sassone-Corsi et al., 2016)
Plasmids		
pBS34	pBluescriptII KS+ ::[XbaI][PstI]KSAC[PstI][XbaI], $Carb^R$ Kan^R	(Raffatellu et al., 2009)
pCRBlunt II-TOPO	TA-Cloning Vector, Kan^R	Invitrogen
pGP704	*oriR6K mobRP4*, $Carb^R$	(Miller and Mekalanos, 1988)
pHP45Ω	$Strep^R$ $Carb^R$	(Prentki and Krisch, 1984)
pKD3	$Carb^R$ Cm^R	(Datsenko and Wanner, 2000)
pKD4	$Carb^R$ Kan^R	(Datsenko and Wanner, 2000)
pJK611	pKD46::*sacB*	Kelly T. Hughes
pJB10	pGP704::*ybtX* FR1-Cm-FR2, Cm^R, $Carb^R$	This study
pNM3	pCRBlunt II-TOPO::*znuA* FR1+FR2, Kan^R	This study
pNM4	pRDH10::*znuA* FR1-FR2, Cm^R	This study
pNM4::KSAC	pRDH10::*znuA* FR1-KSAC-FR2, Cm^R Kan^R	This study
pRDH10	*oriR6K mobRP4 sacRB*, Cm^R Tet^R	(Kingsley et al., 1999)
pSW172	*oriR101 repA101ts*, $Carb^R$	(Lopez et al., 2012)
pHZE107	pGP704::*irp2* FR1-Tet-FR2, Tet^R $Carb^R$	This study

Supplementary Table 2

**Name**	**Sequence (5' - 3')**	**Source**
znuA FR1-Fw	CACATTGGATCCGTTGTTCAGCACCACGAG	This study

znuA FR1-Rv	TATGCCTCTAGACAAGTCTGGTTTCCCTGG	This study
znuA FR2-Fw	TATGCCTCTAGACACACCGCGTTATGTTGG	This study
znuA FR2-Rv	CACATTGGATCCCGGAACTGTTTCGCCGTC	This study
zupT-Fw	GTAAGAACCCGGATAACAATGATGATGATCATCAGTTATTGTGTAGGCTGGAGCTGCTTC	This study
zupT-Rv	TGTTGCCTTTAGCAATGGGCAACATCTGTCATTATCGTCTATGGGAATTAGCCATGGTCC	This study
LB_ybtX_fwd	gccacctgcagatctgcaggCAGACCCCAGATGCTGAAC	This study
LB_ybtX_rev	gtcacaggtaTCCGCGAATAACACAGAG	This study
CmR_fwd	tattcgcggaTACCTGTGACGGAAGATCACTTCGC	This study
CmR_rev	cagggtactcCTTACGCCCCGCCCTGCC	This study
RB_ybtX_fwd	ggggcgtaagGAGTACCCTGCTCAACCACCTGTCCCTC	This study
RB_ybtX_rev	aattcccgggagagctcgatAGCCGGGCGCTGCTGGCG	This study
ybtX_pres_FW	AAAGAGGGTGAAGTCGACAC	This study
ybtX_pres_RV	GTCTATGCAGTCCTTACCCG	This study
ybtX_LB_FW	CTGACGGAACATAAACGAGC	This study
ybtX_RB_RV	TACAGGTGGTGGTGTTATCC	This study
Gib_out_LB_FW	CAATTTGCATAATCGCGTTCAG	This study
Gib-out-RB-RV	AGGGGATGATGGTATTGCGC	This study
ybtX_RED_FW	CACTGATTTTCATCCCTTACCTCTCTGTGTTATTCGCGGAGTGTAGGCTGGAGCTGCTTC	This study
ybtX_LB_FW	CTGGTGATGGAAGAGGGACAGGTGGTTGAGCAGGGTACTCCATATGAATATCCTCCTTA	This study

Gib-out-RB-RV	AGGGGATGATGGTATTGCGC	This study
ybtX_RED_FW	CACTGATTTTCATCCCTTACCTCTCTGTGTTATTCGCGGAGTGTAGGCTGGAGCTGCTTC	This study
ybtX_LB_FW	CTGGTGATGGAAGAGGGACAGGTGGTTGAGCAGGGTACTCCATATGAATATCCTCCTTA	This study
TetR-F	TTAAGACCCACTTTCACATTTAAG	This study
TetA-R	CTAAGCACTTGTCTCCTGTTTAC	This study
pGP704-salI US irp2 F	acctgcagatctgcaggtcgacTCAGTCTGGTGCTGGATG	This study
TetR-US irp2 R	AATGTGAAAGTGGGTCTTAATCTTCCTCCTGATGGCACG	This study
TetA-DS irp2 F	AACAGGAGACAAGTGCTTAGCGCGAAGCAAACTGATTTTCC	This study
pGP704-sacI DS-irp2 R	tcgaattcccgggagagctcTGGCAATATAGTCTTTATCATTG	This study
irp2up-TetRA F	ATGCTTTTCGGTAAGACGTGCCATCAGGAGGAAGATTAAGACCCACTTTCACATTTAAG	This study
irp2dn-TetRA R	GATGGCGTTCCGGGGAAAATCAGTTTGCTTCGCGCTAAGCACTTGTCTCCTGTTTAC	This study
irp2-inF	ACAACGCTTCCTCGTACAATG	This study
Irp2-inR	TCTGTTCGAGCACCTGTTGC	This study
Irp2-upF	TTTGGCGTAACTCCTTCGAC	This study
Irp2-dnR	TTCATAAAGTCAATGGAACG	This study

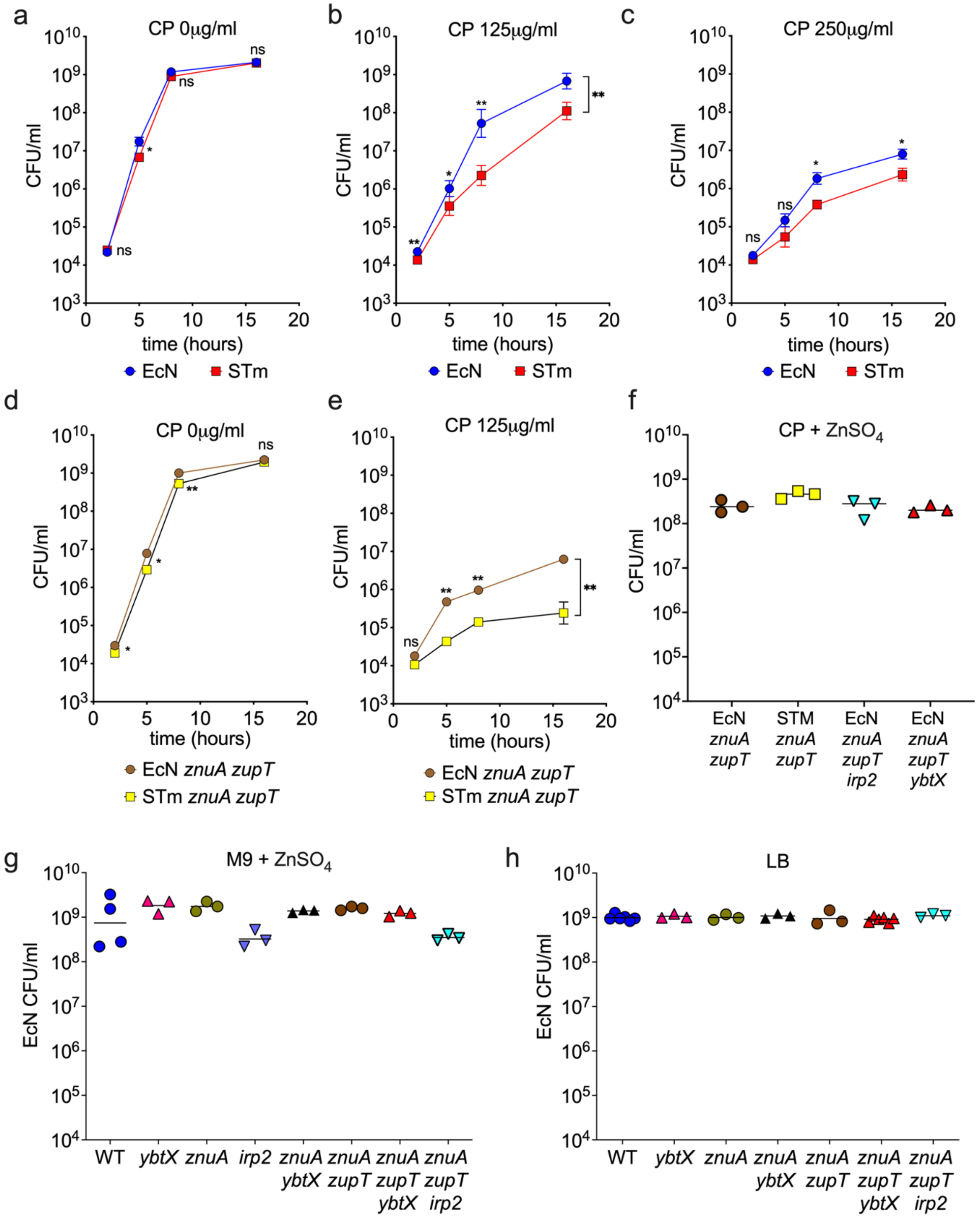
a
CP 0μg/ml
b
CP 125μg/ml
c
CP 250μg/ml
CFU/ml
time (hours)
EcN
STm
ns
d
CP 0μg/ml
e
CP 125μg/ml
EcN znuA zupT
STm znuA zupT
f
CP + ZnSO4
EcN znuA zupT
STM znuA zupT
EcN znuA zupT irp2
EcN znuA zupT ybtX
g
M9 + ZnSO4
EcN CFU/ml
WT
ybtX
znuA
irp2
znuA ybtX
znuA zupT
znuA zupT ybtX
znuA zupT irp2
h
LB

Supplementary Figure 1. Wild-type and mutant strains grow to similar abundance in rich media or in zinc-limiting media supplemented with zinc.

(**a-f**) EcN and STm strains were grown in modified LB medium containing 0, 125, 250 or 150 µg/ml CP and 5 µM $ZnSO_4$ for 16 h static incubation at 37 °C. For **a-e** samples were taken 2 h, 5 h, 8 h and 16 h, and for **f** 16 h after inoculation. Two-sided one sample *t* test was used on log transformed data to accept or reject null hypothesis (theoretical mean = 0). For **a-c**, EcN n = 4 biologically independent replicates, STm n = 3 biologically independent replicates. For **d-f,** n = 3 biologically independent replicates. (**g**) EcN strains were grown in M9 medium supplemented with 5 µM $ZnSO_4$ for 20 h shaking incubation at 37 °C. Different batches of calprotectin were used for **a-e** compared to **f** and main Figure 1. (**h**) EcN strains were grown in LB medium for 20 h shaking incubation at 37 °C. (**f-h**) Each symbol represents an independent biological replicate. (**a-e**) Data are presented as geometric mean values +/- geometric SD. * *P* value ≤0.05; ** *P* value ≤0.01; ns = not significant. Exact *P* values are reported in Supplementary Data 2. Source data are provided as a Source Data file.

a

b

c

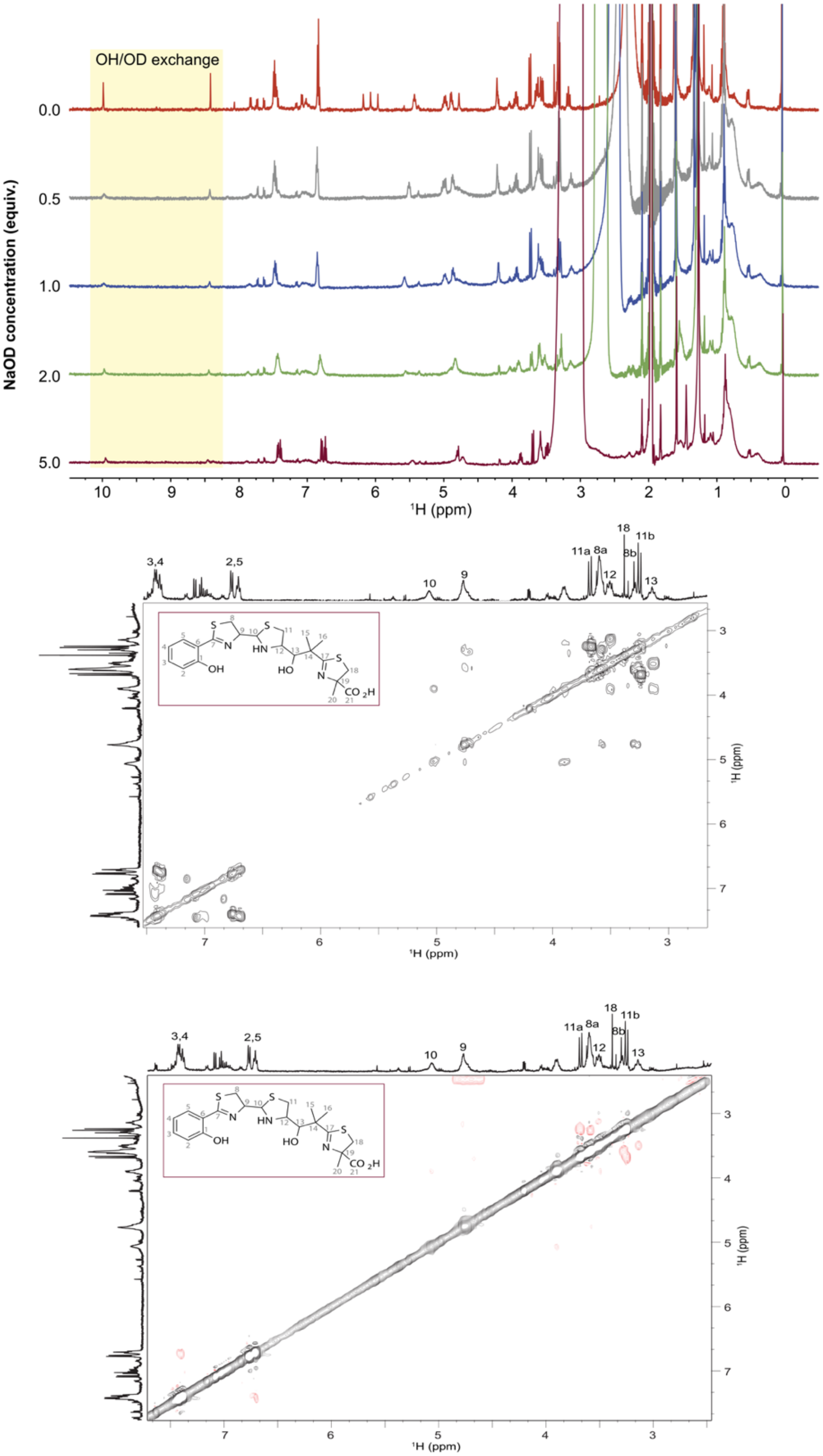
OH/OD exchange
NaOD concentration (equiv.)
0.0
0.5
1.0
2.0
5.0
¹H (ppm)
3,4
2,5
10
9
11a
8a
18
8b
11b
12
13
OH
HN
HO
CO₂H

Supplementary Figure 2. Verification of NMR resonance assignments of Ybt by 2D NMR, and lack of significant changes in Ybt resonances upon the addition of base. (**a**) ^{1}H NMR 1D spectra of Ybt dissolved in CD_3CN in the presence of 5.0 equiv. of $ZnCl_2$ (top trace, 0 equiv. of NaOD, red trace) and increasing amounts of NaOD (0.5 equiv., gray trace), (1.0 equiv., blue trace), (2.0 equiv., green trace), and (5.0 equiv., brown trace). There are not changes in the spectrum other than the loss of OH signal intensity upon addition of NaOD (highlighted in yellow), which is attributed to increased solvent exchange. The starting spectrum of Ybt in the presence of 5.0 equiv. $ZnCl_2$ shows hydrolysis products (signal at 6.2 ppm) in addition to bound and unbound Ybt isomers. (**b**) 2D gradient-enhanced ^{1}H-^{1}H COSY showing aliphatic and aromatic region of spectrum for Ybt dissolved in CD_3CN. (**c**) 2D gradient-enhanced ^{1}H-^{1}H ROESY showing aliphatic and aromatic region of spectrum for Ybt dissolved in CD_3CN. All spectra were acquired at 500 MHz.

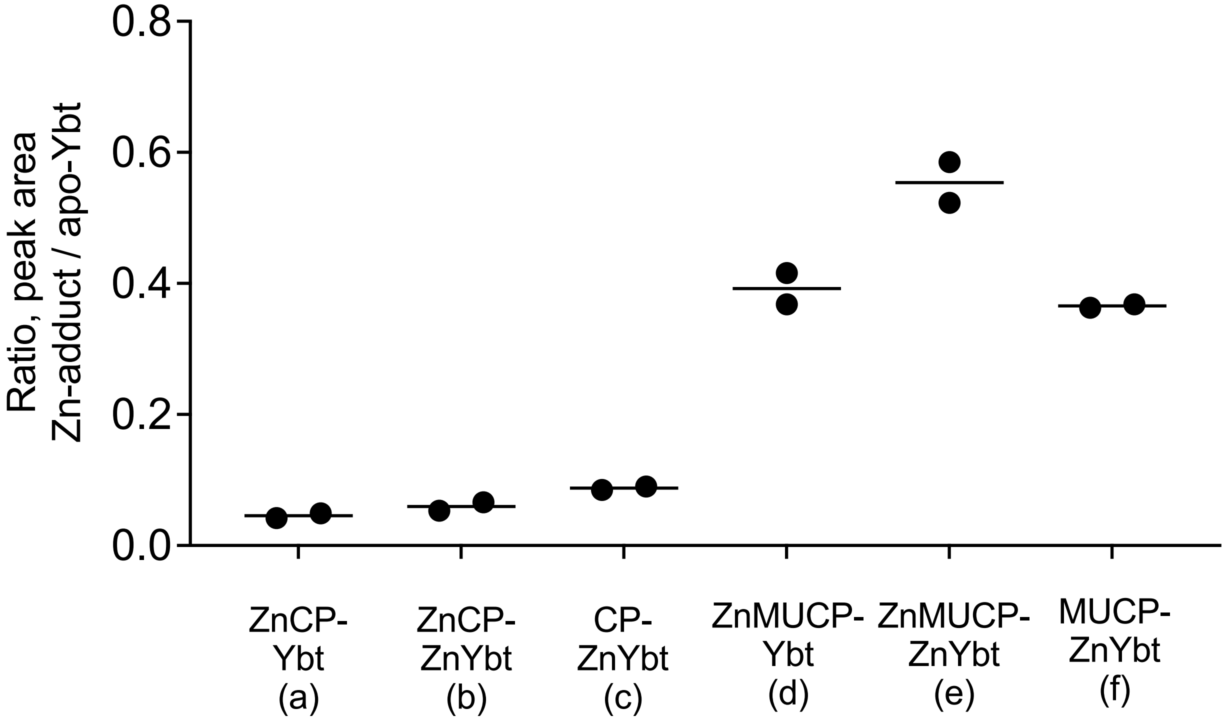

Supplementary Figure 3. **Direct-injection mass spectrometry competition experiments address whether calprotectin (CP) and yersiniabactin (Ybt) exchange zinc.** Competition was carried out between (a) zinc-CP and apo-Ybt, (b) zinc-CP and zinc-Ybt, (c) CP and zinc-Ybt. Control experiments were carried out with mutant-CP (mutCP), which is unable to bind zinc, in place of CP in the following (d) zinc-mutCP and Ybt, (e) zinc-mutCP and zinc-Ybt, and (f) mutCP and zinc-Ybt. Zinc-CP = pre-incubated 0.8 eq Zn and CP-zinc binding site, zinc-Ybt = preincubated with 1eq Zn, zinc-mutCP = preincubated 0.8 eq of Zn with 1 eq mutated CP. Source data are provided as a Source Data file.

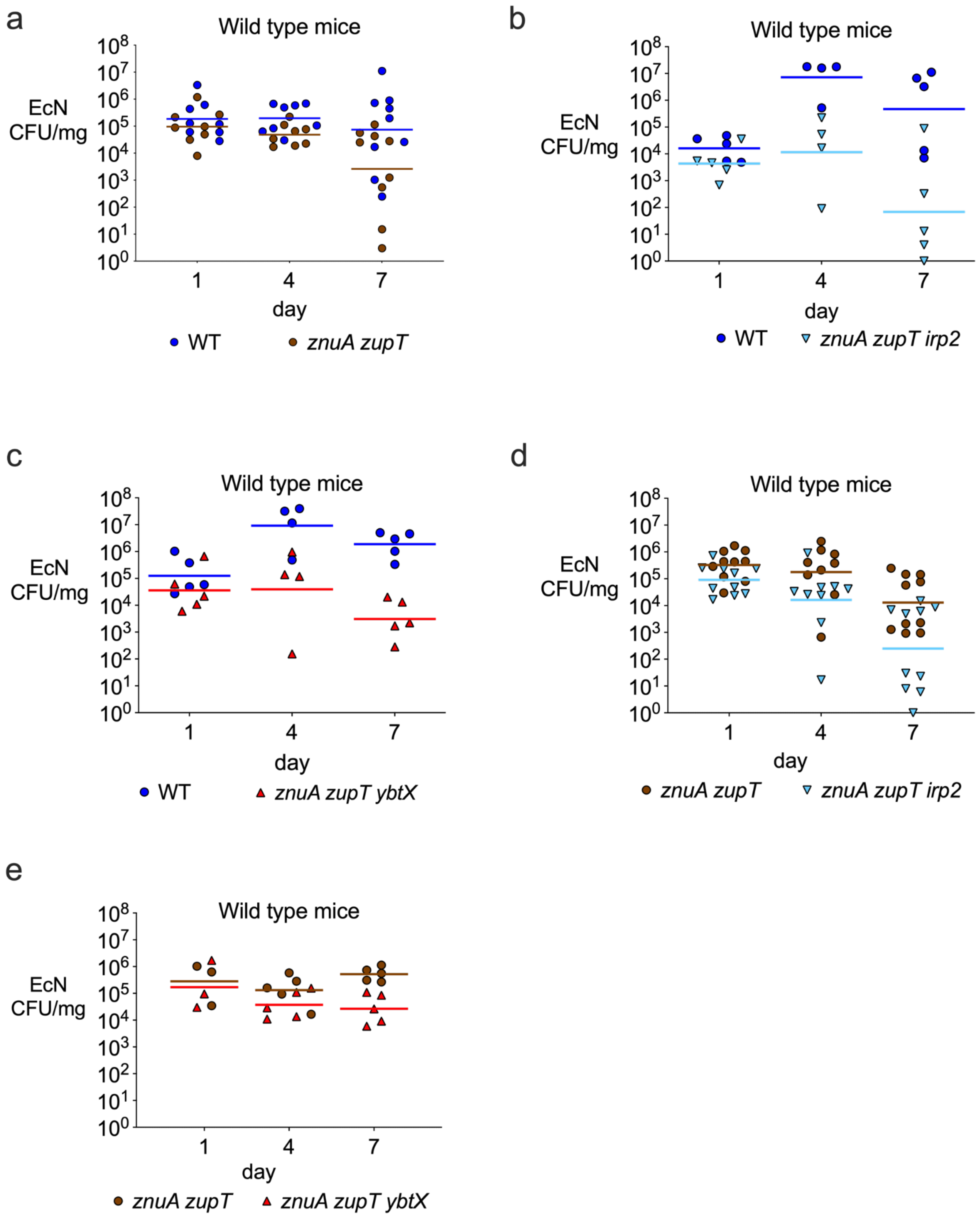
a
Wild type mice
EcN CFU/mg
day
WT
znuA zupT
b
Wild type mice
EcN CFU/mg
day
WT
znuA zupT irp2
c
Wild type mice
EcN CFU/mg
day
WT
znuA zupT ybtX
d
Wild type mice
EcN CFU/mg
day
znuA zupT
znuA zupT irp2
e
Wild type mice
EcN CFU/mg
day
znuA zupT
znuA zupT ybtX

Supplementary Figure 4. EcN fecal CFU from mice in Figure 4.

(**a-e**) CFU of EcN strains in the fecal content of wild-type C57BL/6 mice treated with DSS, all inoculated with the indicated 1:1 mixtures of EcN strains. Dark blue bars represent the geometric mean of EcN wild-type in **a-c**; brown bars represent the geometric mean of the *znuA zupT* mutant in **a**, **d, e**; light blue bars represent the geometric mean of the *znuA zupT irp2* mutant in **b, d**; red bars represent the geometric mean of the *znuA zupT ybtX* mutant in **c, e**. Each symbol represents data from a biologically independent fecal sample from a single mouse. Source data are provided as a Source Data file.

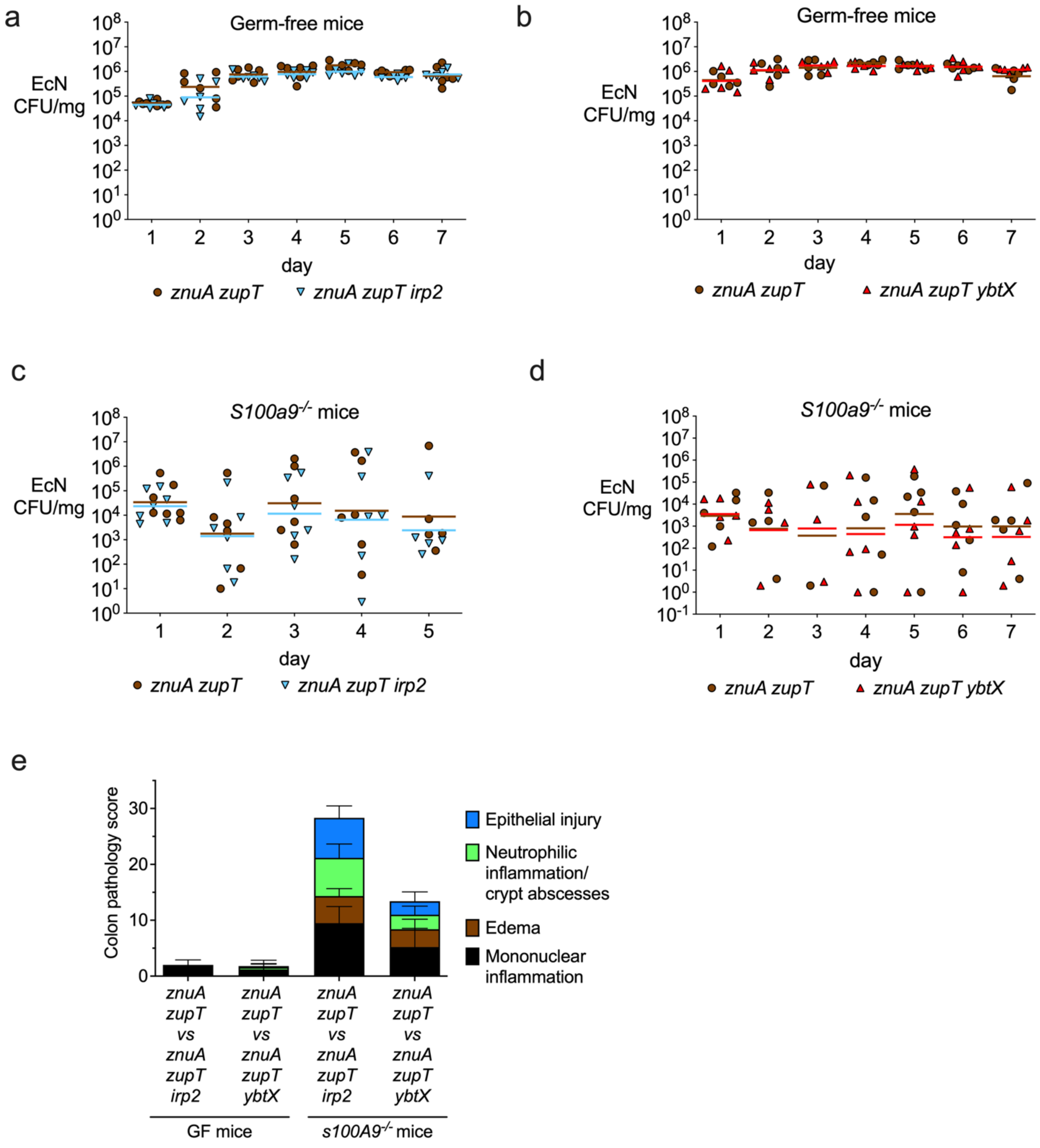
a
Germ-free mice
EcN CFU/mg
day
znuA zupT
znuA zupT irp2
b
Germ-free mice
EcN CFU/mg
day
znuA zupT
znuA zupT ybtX
c
S100a9-/- mice
EcN CFU/mg
day
znuA zupT
znuA zupT irp2
d
S100a9-/- mice
EcN CFU/mg
day
znuA zupT
znuA zupT ybtX
e
Colon pathology score
Epithelial injury
Neutrophilic inflammation/ crypt abscesses
Edema
Mononuclear inflammation
znuA zupT vs znuA zupT irp2
znuA zupT vs znuA zupT ybtX
znuA zupT vs znuA zupT irp2
znuA zupT vs znuA zupT ybtX
GF mice
s100A9-/- mice

Supplementary Figure 5. EcN fecal CFU and pathology scores from mice in Figure 5.

(**a-d**) CFU of EcN strains in the fecal content of (**a, b**) wild-type germ-free Swiss Webster mice or (**c, d**) C57BL/6 *S100a9*$^{-/-}$ mice, all inoculated with the indicated 1:1 mixtures of EcN strains. Brown bars represent the geometric mean of the *znuA zupT* mutant in each experiment; light blue bars represent the geometric mean of the *znuA zupT irp2* mutant in **a, c**; red bars represent the geometric mean of the *znuA zupT ybtX* mutant in **b, d**. Each symbol represents data from a biologically independent fecal sample from a single mouse. (**e**) Pathology scores of colonic tissue from germ-free mice in panel **a, b** (day 7) or of colonic tissue from *S100a9*$^{-/-}$ DSS-treated mice in panel **c** (day 5) and panel **d** (day 7). Germ-free EcN *znuA zupT* vs *znuA zupT irp2,* n = 6; germ-free EcN *znuA zupT* vs *znuA zupT ybtX*, n = 5; *S100a9*$^{-/-}$ mice EcN *znuA zupT* vs *znuA zupT irp2,* n = 6; *S100a9*$^{-/-}$ mice EcN *znuA zupT* vs *znuA zupT ybtX*, n = 5; all colon samples are biologically independent. Data are presented as mean values +/- SD. Source data are provided as a Source Data file.

Supplementary References

1. Herrero, M., de Lorenzo, V., and Timmis, K.N. (1990). Transposon vectors containing non-antibiotic resistance selection markers for cloning and stable chromosomal insertion of foreign genes in gram-negative bacteria. *J. Bacteriol*. *172*, 6557–6567.

2. Platt, R., Drescher, C., Park, S.K., and Phillips, G. J. (2000). Genetic systemfor reversible integration of DNA constructs and LacZ gene fusions into the *Escherichia coli* chromosome. *Plasmid*. *43*, 12-23.

3. Grant, S.G., Jessee, J., Bloom, F.R., and Hanahan, D. (1990). Differential plasmid rescue from transgenic mouse DNAs into *Escherichia coli* methylation-restriction mutants. *Proc. Natl. Acad. Sci. U. S. A*. *87*, 4645–4649.

4. Stojiljkovic, I., Baumler, A.J., and Heffron, F. (1995). Ethanolamine utilization in *Salmonella typhimurium*: nucleotide sequence, protein expression, and mutational analysis of the *cchA cchB eutE eutJ eutG eutH* gene cluster. *J. Bacteriol*. *177*, 1357–1366.

5. J. Z. Liu, S. Jellbauer, A. Poe, M. Pesciaroli, T. Kehl-Fie, N. A. Restrepo, M. Hosking, R. A. Edwards, A. Battistoni, P. Pasquali, T. E. Lane, W. Chazin, T. Vogl, J. Roth, E. P. Skaar, and M. Raffatellu. Zinc sequestration by the neutrophil protein calprotectin enhances *Salmonella* growth in the inflamed gut. *Cell Host and Microbe*. (2012) March; *11* (3); 227-239

6. M. Cerasi, J. Z. Liu, S. Ammendola, A. J. Poe, P. Petrarca, M. Pesciaroli, P. Pasquali, M. Raffatellu, A. Battistoni. The ZupT transporter plays a central role in zinc homeostasis and contributes to *Salmonella enterica* virulence. *Metallomics***.** (2014) Mar 26;6(4):845-53

7. M. Sassone-Corsi, S. Nuccio, D. Hernandez, H. Liu, C. T. Vu, A. A. Takahashi, R. A. Edwards, and M. Raffatellu. Microcins mediate competition among Enterobacteriaceae in the inflamed gut. *Nature*. 2016. Dec 8;540(7632):280-283

8. M. Raffatellu, M.D. George, Y. Akiyama, M.J. Hornsby, S. Nuccio, T. A. Paixão, B. P. Butler, H. Chu, R. L. Santos, T. Berger, T. W. Mak, R.M. Tsolis, C.L. Bevins, J. V. Solnick, S. Dandekar and A.J. Bäumler. Lipocalin-2 resistance confers an advantage to *Salmonella enterica* serotype Typhimurium for growth and survival in the inflamed intestine. *Cell Host and Microbe*. (2009) 5(5): 476-486.

9. Miller, V.L., and Mekalanos, J.J. (1988). A novel suicide vector and its use in construction of insertion mutations: osmoregulation of outer membrane proteins and virulence determinants in *Vibrio cholerae* requires *toxR*. *J. Bacteriol*. *170*, 2575–2583.

10. Prentki, P., and Krisch, H.M. (1984). In vitro insertional mutagenesis with a selectable DNA fragment. *Gene 29*, 303–313.

11. Datsenko, K.A., and Wanner, B.L. (2000). One-step inactivation of chromosomal genes in *Escherichia coli* K-12 using PCR products. *Proc. Natl. Acad. Sci. U. S. A*. *97*, 6640–6645.

12. Kingsley, R.A., Reissbrodt, R., Rabsch, W., Ketley, J.M., Tsolis, R.M., Everest, P., Dougan, G., Bäumler, A.J., Roberts, M., and Williams, P.H. (1999). Ferrioxamine-mediated Iron(III) utilization by *Salmonella enterica*. *Appl. Environ. Microbiol*. *65*, 1610–1618.

13. Lopez, C.A., Winter, S.E., Rivera-Chávez, F., Xavier, M.N., Poon, V., Nuccio, S.-P., Tsolis, R.M., and Bäumler, A.J. (2012). Phage-mediated acquisition of a type III secreted effector protein boosts growth of *Salmonella* by nitrate respiration. *MBio 3*. Jun 12;3(3):e00143-12
