# Supplementary material for: Siderophore-mediated zinc acquisition enhances enterobacterial colonization of the inflamed gut
Source: Nat Commun. 2021 Dec 1;12:7016. doi: 10.1038/s41467-021-27297-2 (PMC8636617; doi:10.1038/s41467-021-27297-2)
Supplement: Supplementary file 2 — Description of Additional Supplementary Files [file 41467_2021_27297_MOESM2_ESM.pdf]

File name: Supplementary Data 1

Description: Genomic variations among strains used in this study

File name: Supplementary Data 2

Description: Calculated P values
