# Supplementary material for: Siderophore-mediated zinc acquisition enhances enterobacterial colonization of the inflamed gut
Source: Nat Commun. 2021 Dec 1;12:7016. doi: 10.1038/s41467-021-27297-2 (PMC8636617; doi:10.1038/s41467-021-27297-2)
Supplement: Supplementary file 5 — Reporting Summary [file 41467_2021_27297_MOESM5_ESM.pdf]

nature portfolio

Corresponding author(s): Manuela Raffatellu

Last updated by author(s): Oct 24, 2021

Reporting Summary

Nature Portfolio wishes to improve the reproducibility of the work that we publish. This form provides structure for consistency and transparency in reporting. For further information on Nature Portfolio policies, see our Editorial Policies and the Editorial Policy Checklist.

Statistics

For all statistical analyses, confirm that the following items are present in the figure legend, table legend, main text, or Methods section.

n/a	Confirmed	
☐	☒	The exact sample size (*n*) for each experimental group/condition, given as a discrete number and unit of measurement
☐	☒	A statement on whether measurements were taken from distinct samples or whether the same sample was measured repeatedly
☐	☒	The statistical test(s) used AND whether they are one- or two-sided<br>*Only common tests should be described solely by name; describe more complex techniques in the Methods section.*
☐	☒	A description of all covariates tested
☐	☒	A description of any assumptions or corrections, such as tests of normality and adjustment for multiple comparisons
☐	☒	A full description of the statistical parameters including central tendency (e.g. means) or other basic estimates (e.g. regression coefficient) AND variation (e.g. standard deviation) or associated estimates of uncertainty (e.g. confidence intervals)
☐	☒	For null hypothesis testing, the test statistic (e.g. *F*, *t*, *r*) with confidence intervals, effect sizes, degrees of freedom and *P* value noted<br>*Give P values as exact values whenever suitable.*
☒	☐	For Bayesian analysis, information on the choice of priors and Markov chain Monte Carlo settings
☒	☐	For hierarchical and complex designs, identification of the appropriate level for tests and full reporting of outcomes
☒	☐	Estimates of effect sizes (e.g. Cohen's *d*, Pearson's *r*), indicating how they were calculated

Our web collection on statistics for biologists contains articles on many of the points above.

Software and code

Policy information about availability of computer code

Data collection	Mass Spectrometry: Thermo XCalibur software and Tune
Data analysis	Statistical Analysis: Prism 9.<br>Molecular Networking: MZMine 2.37corr17.7_kai_merge2, GNPS, SIRIUS 4.0.1 (build 9)<br>Genome Sequence Analysis: FastQC, BWA MEM, Picard MarkDuplicates, freeBayes, Annovar

For manuscripts utilizing custom algorithms or software that are central to the research but not yet described in published literature, software must be made available to editors and reviewers. We strongly encourage code deposition in a community repository (e.g. GitHub). See the Nature Portfolio guidelines for submitting code & software for further information.

Data

Policy information about availability of data

All manuscripts must include a data availability statement. This statement should provide the following information, where applicable:

- Accession codes, unique identifiers, or web links for publicly available datasets
- A description of any restrictions on data availability
- For clinical datasets or third party data, please ensure that the statement adheres to our policy

Source data are provided with this paper. All NMR raw data are provided. For genome analysis, we used the E. coli Nissle 1917 wild-type strain reference genome (GenBank "CP022686.1 [https://www.ncbi.nlm.nih.gov/nucleotide/CP022686.1]"). All mass spectrometry .raw and centroid .mzXML or .mzML files, in addition to MZmine 2 outputs and project file, are publicly available in the mass spectrometry interactive virtual environment (MassIVE) under massive.ucsd.edu with project identifier MSV000083387 (E. coli Nissle siderophores); raw spectra of yersiniabactin commercial standards are available under MSV000084237 (Siderophore Standard Mixture with metal additions). Ion Identity Molecular Networks can be accessed through gnps.ucsd.edu under direct links:

https://gnps.ucsd.edu/ProteoSAFe/status.jsp?task=525fd9b6a9f24455a589f2371b1d9540 and http://gnps.ucsd.edu/ProteoSAFe/status.jsp?task=e2bd16458ec34f3f9f99982dedc7d158.

Field-specific reporting

Please select the one below that is the best fit for your research. If you are not sure, read the appropriate sections before making your selection.

☒ Life sciences ☐ Behavioural & social sciences ☐ Ecological, evolutionary & environmental sciences

For a reference copy of the document with all sections, see nature.com/documents/nr-reporting-summary-flat.pdf

Life sciences study design

All studies must disclose on these points even when the disclosure is negative.

Sample size	For the in vitro growth assays, the variation is minimal and an n=3 independent biological replicates or larger was used. We chose this sample size based on our prior experience of the number of experiments needed to detect a 10-fold difference. For the animal experiments, the sample size was determined based on the fact that, in mixed infection, every mouse has an internal control (i.e., we are comparing two strains in a given animal). Based on our experience and prior publications, an n=5 mice is sufficient to determine whether one strain has a competitive advantage over another strain in vivo. The differences between wild-type and mutants are quite large (from ~20-fold to ~10,000-fold) and can be detected with n=5 mice.
Data exclusions	No data were excluded from the analysis.
Replication	All experiments were performed at least twice to ensure reproducibility. We obtained similar results at three different institutions (UC Irvine, UC San Diego, University of Illinois at Chicago), and all attempts of replication were successful.
Randomization	Mice were randomly assigned to groups. For other experiments, randomization was not relevant (e.g. bacterial growth curves were performed by inoculating known bacteria to relevant media that was aliquoted in wells).
Blinding	Blinding was only performed with histopathology, as it is by nature a subjective/qualitative assessment of samples. The pathologist was blinded to group allocation during data collection. All other data generation and analysis were not blinded, but were performed using agnostic protocols and procedures (e.g., orogastric gavage) with objective modes of data acquisition (e.g., counting colonies on an agar surface).

Reporting for specific materials, systems and methods

We require information from authors about some types of materials, experimental systems and methods used in many studies. Here, indicate whether each material, system or method listed is relevant to your study. If you are not sure if a list item applies to your research, read the appropriate section before selecting a response.

Materials & experimental systems

n/a	Involved in the study
☒	☐ Antibodies
☒	☐ Eukaryotic cell lines
☒	☐ Palaeontology and archaeology
☐	☒ Animals and other organisms
☒	☐ Human research participants
☒	☐ Clinical data
☒	☐ Dual use research of concern

Methods

n/a	Involved in the study
☒	☐ ChIP-seq
☒	☐ Flow cytometry
☒	☐ MRI-based neuroimaging

Animals and other organisms

Policy information about studies involving animals; ARRIVE guidelines recommended for reporting animal research

Laboratory animals	Germ-free Swiss Webster mice as well as specific pathogen-free C57BL/6 wild-type mice and S100a9-/- mice were used in our study. C57BL/6 mice were purchased from Jackson Lab, whereas S100a9-/- mice (Manitz et al., 2003) were bred in-house. Mice at UC Irvine and at UC San Diego were fed diet Teklad 2920X. Germ-free Swiss Webster mice were purchased from Taconic Farms and then bred in-house in germ-free isolators (Park Bio). These mice were fed irradiated diet Purina 5066. We used both male and female mice. The mice were kept in a 12h light/dark cycle, at a room temperature of ~22 °C and ~52% humidity.
Wild animals	No wild animals were used in the study
Field-collected samples	No field collected samples were used in the study.

Ethics oversight

All experiments were performed in accordance with approved Institutional Animal Care and Use Committee protocols and guidelines of the University of California, Irvine, the University of California, San Diego, and the University of Illinois at Chicago.

Note that full information on the approval of the study protocol must also be provided in the manuscript.
